# Synthesis of New Isoxazolidine Derivatives Utilizing the Functionality of *N*-Carbonylpyrazol-Linked Isoxazolidines

**DOI:** 10.3390/molecules29153454

**Published:** 2024-07-23

**Authors:** Xixian Cao, Jun You, Yunze Wang, Yanchao Yu, Wenju Wu, Yifang Liang

**Affiliations:** Key Laboratory of Green Chemical Engineering and Technology of Heilongjiang Province, College of Materials Science and Chemical Engineering, Harbin University of Science and Technology, Harbin 150080, China; caoxixian1001@163.com (X.C.); wangyunze01@163.com (Y.W.); yychao136@163.com (Y.Y.); wuwenju1017@163.com (W.W.)

**Keywords:** 3,5-dimethylpyrazole, isoxazolidine, 1,3-dipolar cycloaddition, nucleophilic substitution, reduction reaction

## Abstract

Using Ni(II) as the catalyst, electron-deficient 3,5-dimethylacryloylpyrazole olefin was reacted with *C*,*N*-diarylnitrones alone for 10 min to prepare novel five-member heterocyclic products, 4-3,5-dimethylacryloylpyrazole isoxazolidines with 100% regioselectivity and up to 99% yield. And then, taking these cycloadducts as substrates, six kinds of derivatization reactions, like ring-opening, nucleophilic substitution, addition-elimination and reduction, were studied. Experimental results showed that all kinds of transformations could obtain the target products at a high conversion rate under mild conditions, a finding which provided the basic methods for organic synthesis methodology research based on an isoxazolidine skeleton.

## 1. Introduction

Isoxazolidine compounds are a part of the azole family and contain O and N atoms in the 1,2-positions; these compounds serve as influential building blocks for bioactive molecules and natural products [1,2]. Also, isoxazolidine compounds are often utilized as multipurpose intermediates in research subjects as diverse as organic synthesis, medicinal chemistry and material science [3,4,5]. Among the existing methods, the 1,3-dipolar cycloaddition of nitrone with olefin is an important method used to gain structurally diverse isoxazolidines [6,7]. The molecular structure of nitrones and alkenes plays a key role in the rate, selectivity and conversion of the cycloaddition reaction; therefore, selecting auxiliary groups with good inducing functions for nitrones and alkenes is of great significance. On the other hand, as can be seen from the structural characteristics of olefin, the leaving activity of the olefin auxiliary group is all-important in researching whether other active functional groups can be easily introduced into the isoxazolidine rings.

A previous work reported by our group showed that 3,5-dimethylpyrazolylcarbonyl olefin is an excellently reactive electron-deficient olefin in the 1,3-dipole cycloaddition reaction [8]. However, (pyrazolylcarbonyl)isoxazolidine derivatizations are rarely systematically studied. In view of this, we attempted to modify isoxazolidine intermediates by employing 3,5-dimethylpyrazolylcarbonyl as an acylating reagent in the study of isoxazolidine derivatization.

**Figure 1:**
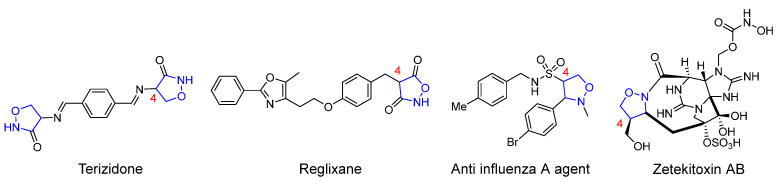
Drugs and natural products containing 4-substituted isoxazolidine rings.

Due to the outstanding performance of the isoxazolidine structural fragment in various fields, the construction of novel isoxazolidine derivants through simple and effective synthetic methods has long been a hot topic among organic synthetic chemists. In general, most of the works investigating isoxazolidines-involved derivatization reactions focus on the study of ring-opening reduction to form *β*-alkamine, and the rearrangement to form amino alcohols and preparation as lactams [9,10,11,12]. Meanwhile, the 4-isoxazolidine derivatives with varying substituents exemplify an important way of increasing the application value of these compounds, since they retain the heterocyclic skeletons and also provide reaction sites in synthetic chemistry [13,14]. Figure 1 shows the 4-substituted isoxazolidine derivatives in several drug molecules or natural products [15,16,17,18].

**Figure 2:**
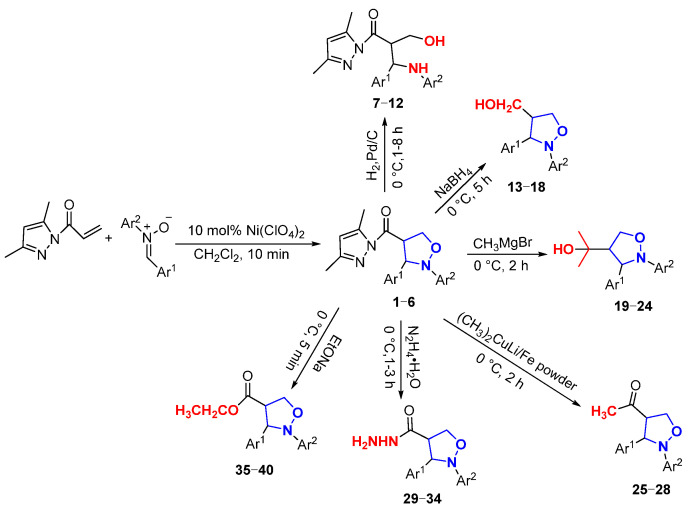
The synthesis routes of isoxazolidines and their further derivatization reactions.

In this paper, novel 4-functionalized isoxazolidines attaching a 3,5-dimethylpyrazolylcarbonyl group with 100% regioselectivity and up to 99% yield were synthesized through a 1,3-dipolar cycloaddition process of *C*,*N*-diarylnitrones to 3,5-dimethylacryloylpyrazole alkene using a Ni (II) catalyst. Subsequently, we exploited six kinds of transformations, including ring-opening, nucleophilic substitution, and reduction, to construct a series of new 4-substituted isoxazolidine derivatives with moderate to excellent yields under mild conditions; the specific routes are shown in Figure 2.

## 2. Results and Discussion

**Figure 3:**
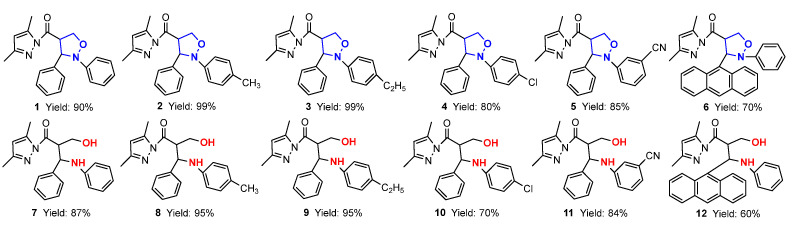

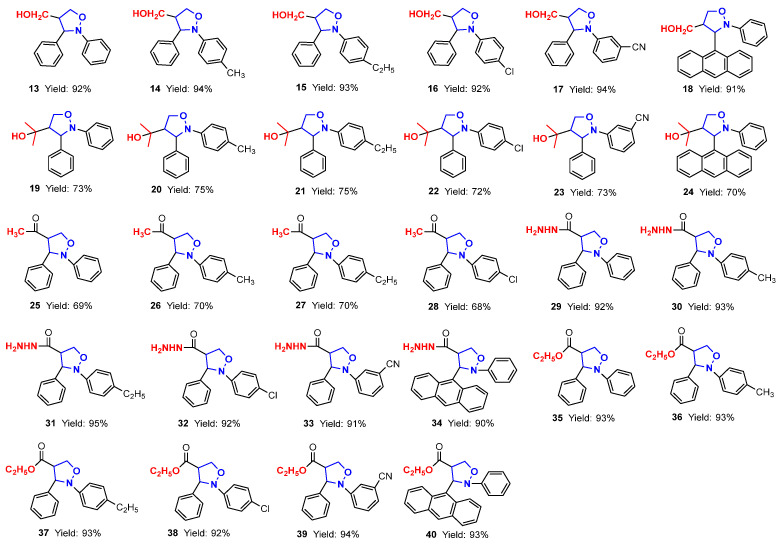
The structures and yields of isoxazolidines and their derivatives.

In this paper, firstly, 4-(3,5-dimethylpyrazol-1-ylcarbonyl) isoxazolidines (**1**–**6**) were synthesized, and secondly, six kinds of isoxazolidine derivatives (**7**–**40**) were synthesized from these ingredients. The structures and yields of the isoxazolidines and their derivatives are shown in Figure 3.

### 2.1. Synthesis of 2,3-Diaryl-4-(3,5-dimethylpyrazolylcarbonyl)isoxazolidines ***1***–***6***

For the 1,3-dipolar cycloaddition reaction of *C,N*-diarylnitrones with 3,5-dimethylacryloylpyrazole olefin studied in this paper, the reaction took only 10 min to give 4-substituted isoxazolidine products with 100% regioselectivity when using 10 mol% Ni(ClO_4_)_2_·6H_2_O·as catalyst; the structure of 2-phenyl-3-(9-anthryl)-4-(3,5-dimethylpyrazolylcarbonyl)isoxazolidine (**6**) was confirmed by single-crystal X-ray diffraction (Table 1). The effects of different groups of nitrones on the 1,3-dipolar cycloaddition were probed. When there was no substituent on the benzene ring Ar^2^ of the N-atom, the yield of cycloadduct **1** reached 90% for 10 min (Entry 1). When the benzene ring of Ar^2^ contained electron-donating groups (-CH_3_ and -C_2_H_5_), the yields of **2** and **3** reached 99% (Entries 2–3). However, when it contained electron-withdrawing groups (-Cl and -CN), the yields of **4** and **5** dropped to 80% and 85%, respectively (Entries 4–5). Additionally, when the benzene ring of Ar^1^ was replaced by bulky 9-anthryl substituents, the cycloaddition went smoothly and the yield of **6** was 70% at the same reaction time (Entry 6). The results showed that different substituents on the benzene ring Ar^2^ of *C*,*N*-diarylnitrones had good substrate universality relative to the product conversion.

### 2.2. Study of the Reduction Reactions of (Pyrazolylcarbonyl)isoxazolidines

**Figure 4:**
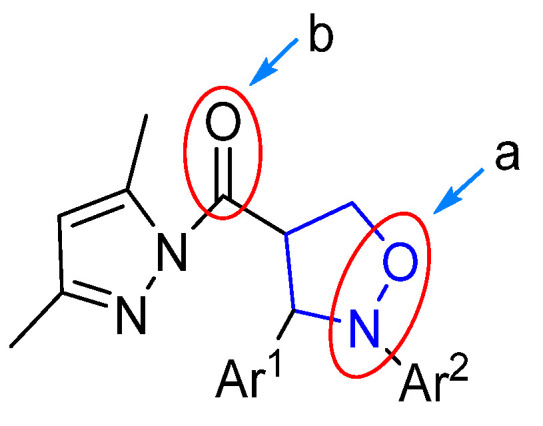
Reduction position of (pyrazolylcarbonyl)isoxazolidine.

As depicted in Figure 4, the (pyrazolylcarbonyl)isoxazolidine-derived molecule had two easily reducible reactive sites (**a** and **b**), which created the possibility of the preparation of diverse isoxazolidine derivatives. Currently, the study of isoxazolidine derivatizations is mainly focused on the ring-opening hydrogenation reduction (in the site **a**) to acquire *β*-alkamine with the aid of suitable reducing agents. Compared to the isoxazolidine ring, due to the high reactivity of the pyrazolylcarbonyl, the carbonyl group in this structure is more prone to reduction reactions. Therefore, the methodology of choosing the appropriate reducing agent and selectively opening the isoxazolidine ring while retaining the pyrazole group to prepare new *β*-alkamines or retaining the isoxazolidine ring to prepare the 4-position carbonyl group-reduced products is a challenging task. Some known samples have revealed ring-opening reduction catalysts like Raney-Ni [19], Zn/H^+^ [20], Mo(CO)_6_ [21] and Pd/C [22,23], which have catalyzed ring-opening reductions of isoxazolidine to generate *β*-alkamine. The experiment described, however, found that only the 3,5-dimethylpyrazolyl group was unaffected within the method of Pd/C catalyst hydrogenation. Given this, the effects of different groups of (pyrazolylcarbonyl)isoxazolidines on the reduction reaction were investigated by the use of a Pd/C catalyst.

As for the ring-opening hydrogenation reduction of (pyrazolylcarbonyl)isoxazolidines, Table 2 showed that the differences in the groups of (pyrazolylcarbonyl)isoxazolidines had a relatively large influence, causing significant differences in the reaction times and product yields. The yield of product **7** was 87% for 2 h when the benzene ring Ar^2^ of the N-atom had no substituent (Entry 1). When the benzene ring of Ar^2^ contained electron-donating substituents (-CH_3_ and -C_2_H_5_), the yields of **8** and **9** were distinctly increased to 95%, although the reaction time was shortened to 1 h (Entries 2–3). When the benzene ring of Ar^2^ contained electron-withdrawing groups (-Cl and -CN) and the Ar^1^ substituent was the 9-anthryl group, products **10**, **11** and **12** had, respectively, yields of 70, 84 and 60%, and respective reaction times of 8, 3 and 8 h (Entries 4–6). It is known that 4-hydroxymethylated isoxazolidine derivants have some uses in medicinal chemistry and other subjects [24,25]. Here, the use of sodium borohydride (NaBH_4_) to induce the reduction reaction of isoxazolidines was studied. When the molar ratio of reactant to NaBH_4_ was 1:4, a yield of **13** achieved a maximum value of 92% for 5 h (Entries 7). The effects of substituents of (pyrazolylcarbonyl)isoxazolidines on the reaction were also studied, and the results showed that differences in the substituents had little effect on the reactions under the same conditions (Entries 8–12).

### 2.3. Study of the Nucleophilic Reactions of (Pyrazolylcarbonyl)isoxazolidines

From the perspective of some works in the literature and potential applications [26,27,28], the introduction of four functional groups (-C(CH_3_)_2_OH, -COCH_3_, -CONHNH_2_ and -COOC_2_H_5_) into the 4-position of the isoxazolidine ring was very important. Therefore, the involvement of the 3,5-dimethylpyrazolylcarbonyl group in the derivatization reactions broadened the synthetic utility of the isoxazolidines.

#### 2.3.1. Study of the Nucleophilic Substitution Reactions with Organometallic Reagents

The involvement of (pyrazolylcarbonyl)isoxazolidine in a nucleophilic substitution reaction with Grignard reagent CH_3_MgBr constituted an efficient method to obtain 2-(2-(aryl)-3-arylisoxazolidin-4-yl)propan-2-ol (**19**–**24**) in 70–75% yields at 0 °C for 2 h under argon. To retain a methyl ketone fragment, we tried to lower the amount of CH_3_MgBr and the reaction temperature. However, the stronger leaving property of the 3,5-dimethylpyrazolylcarbonyl group and the stronger reactivity of CH_3_MgBr led to the methyl ketones produced being eventually converted into tertiary alcohols. To address this, CH_3_MgBr was replaced with the less reactive (CH_3_)_2_CuLi, and 4-acetyl-2,3-diarylisoxazolidines (**25**–**28**) with methyl ketone fragments in 65–70% yields were obtained under the same conditions (Table 3).

Initially, the product was generated in the tertiary alcohol stage by controlling the input amount of the CH_3_MgBr. There was hardly any reaction when the amount of CH_3_MgBr was lower than 1 equivalent at 0 °C (Entries 1–2). When the amount of CH_3_MgBr increased to 1, 5 and 10 equivalents, the yields of product **19** with a tertiary alcohol structure reached 7%, 30% and 73% for 2 h, respectively (Entries 3–7). Additionally, the target had an identical yield (73%) for 30 min when using 11-equivalent amounts of CH_3_MgBr (Entry 8). The ingredients disappeared and only a small amount of product was synthesized across a duration of 30 min when the reaction temperature was raised to room temperature (Entry 9), possibly because the hyperalkalinity of CH_3_MgBr caused the decomposition of both the ingredients and the product. Further, **19** had respective yields of only 65% and 52% when the amount of CH_3_MgBr was 11 equivalents and the reaction temperature was further reduced to −10 °C and −20 °C for 3 h (Entries 10–11). Meanwhile, experiment’s results showed that different groups of 4-(pyrazolylcarbonyl)isoxazolidines had little effect on the reaction under the 10-equivalent amounts of CH_3_MgBr for 2 h at 0 °C (Entries 12–16).

4-Acetyl-2,3-diarylisoxazolidines (**25**–**28**) in 68–70% yields were readily prepared when using 3-equivalent amounts of the less reactive (CH_3_)_2_CuLi at 0 °C. Unexpectedly, the reaction could not be reproduced when the experiment was repeated later (Entry 17). We carefully observed and studied the experimental results and found that the polytetrafluoroethylene stir-bar surface exposed a small amount of iron dust in the original reaction system. Simultaneously, we consulted some literature on nucleophilic substitution reactions involving organometallic reagents and found that iron catalysts could catalyze this reaction. Subsequently, we tried using iron powder as catalyst, and the experimental results showed that the reaction could proceed smoothly [29]. Hence, when the molar ratio of (pyrazolylcarbonyl)isoxazolidine and (CH_3_)_2_CuLi was 1:3, acetylation products were obtained with a trace amount of Fe powder catalysis at 0 °C under argon (Entries 18–21).

#### 2.3.2. Study of the Hydrazinolysis and Alcoholysis Reactions of (Pyrazolylcarbonyl)isoxazolidines

To explore the nucleophilicity of (pyrazolylcarbonyl)isoxazolidines toward 80*w*% hydrazine hydrate, hydrazinolysis reactions were performed under mild conditions (Table 4). When there was no substituent in the benzene ring of (pyrazolylcarbonyl)isoxazolidines, the yield of **29** reached 70% at 0 °C for 1 h (Entry 1). The yield of product was reduced to 45% when the reaction temperature was raised to room temperature (Entry 2), likely due to the strong alkalinity of 80*w*% hydrazine hydrate, which led to partial hydrolysis of the (pyrazolylcarbonyl)isoxazolidine [30]. The yield of product was increased to 92% when reaction time was extended to 3 h at 0 °C (Entries 3–4), and the yield of product slightly roset (93% yield) when the reaction time was prolonged to 4 h (Entry 5). When the benzene ring on the N-atom contained electron-donating groups (-CH_3_, -C_2_H_5_), the yields of compounds **30** and **31** reached 93% and 95%, respectively (Entries 6–7). When it contained electron-withdrawing groups (-Cl, -CN), compounds **32** and **33** had respective yields of 92% and 91% (Entries 8–9). And when the benzene ring on Ar^1^ was replaced with a larger volume of 9-anthryl, the yield of **34** reached 90% for 3 h (Entry 10).

Next, to explore the nucleophilicity of (pyrazolylcarbonyl)isoxazolidines toward nucleophiles, the effects of varying nucleophile dosages, reaction temperatures, reaction times and varying groups of isoxazolidines within the alcoholysis reaction were tested (Table 5). Using methods described in the literature [23,31], no products were generated, according to TLC analysis, when using a 0.8 equivalent of sodium methoxide (CH_3_ONa) at room temperature for 30 min (Entry 1), probably because the strong reactivity of CH_3_ONa led to the loss of ingredients thorough decomposition. Products **35′** had respective yields of 60% and 73% when using 0.8 equivalent and 1.1 equivalents of CH_3_ONa at 0 °C for 5 min (Entries 2–3). However, the utilization of 1.1 equivalents of sodium ethoxide (EtONa) further enhanced the yield of **35** (93% yield) at 0 °C for 5 min (Entry 4). With the continuous extension of the reaction time, the quantity of the products would gradually become less until the product almost completely disappeared after 25 min (Entries 5–8). The yield of product was significantly reduced when using less than 1 equivalent of EtONa (Entries 9–10). Consequently, when the molar ratio of (pyrazolylcarbonyl)isoxazolidine with EtONa was 1:1.1, ethyl 2,3-diarylisoxazolidin-4-ylcarboxylate (**35**–**40**) with 92–94% yields was gained at 0 °C for 5 min under argon (Entries 4 and 11–15).

## 3. Experimental Design

### 3.1. Instrumentation and Materials

IR spectra were recorded by a Thermo Nicolet 370 Fourier transform infrared (FTIR) spectrometer, with the samples in KBr pellets. The ^1^H and ^13^C NMR spectra were obtained using a Bruker 300 NMR spectrometer (300 and 75 MHz, respectively) in CDCl_3_. Abbreviations for data cited are as follows: s, singlet; d, doublet; t, triplet; dd, doublet of doublets; m, multiplet. The residual solvent signals were served as references and the chemical shifts converted to the TMS scale (CDCl_3_: δ_H_ = 7.26 ppm, δ_C_ = 77.16 ppm). High-resolution mass spectra were obtained on a Waters G2-XS QTof mass spectrometer. Melting points were determined with a Tektronix X-6 micro-melting-point apparatus and are uncorrected.

All chemicals and reagents were purchased from sellers and employed as received.

### 3.2. Synthesis Methods

The starting materials, specifically, the alkene and nitrones, were obtained according to a previously published method [32].

#### 3.2.1. Synthesis of 2,3-Diaryl-4-(3,5-dimethylpyrazolylcarbonyl)isoxazolidines (**1**–**6**)

Compounds (**1**–**6**) were prepared by the reported procedure [8]. 3,5-dimethylacryloyl pyrazole alkene (447 mg, 2.98 mmol), Ni(ClO_4_)_2_·6H_2_O (109 mg, 0.298 mmol) and isopropanol (2 mL) were added to CH_2_Cl_2_ (20 mL), and, next, a solution of *C*,*N*-diaryl nitrones (646 mg, 3.28 mmol) in CH_2_Cl_2_ (10 mL) was added dropwise. The reaction was stirred for 10 min at room temperature. The solvent was evaporated in a vacuum and the crude products were purified by preparative TLC (eluent: petroleum ether/ethyl acetate = 20/1) to gain the cycloadducts **1**–**6**. Corresponding data and spectra are given in the Supplementary Materials.

#### 3.2.2. Synthesis of 2-(3,5-Dimethylpyrazol-1-ylcarbonyl)-3-(arylamino)-3-arylpropan-1-ol (**7**–**12**)

Compounds **1** or **2**–**6** (50 mg, 0.144 mmol) and 5% Pd/C (25 mg, 0.231 mmol) were added to EA (4 mL), and the mixture was stirred for 1–8 h under a hydrogen atmosphere, while the reaction was monitored by TLC (R_f_ = 0.23 (PE/EA = 5/1). The reaction mixture was purified by preparative TLC (eluent: PE/EA = 5/1) to obtain products **7**–**12**. Corresponding data and spectra are given in the Supplementary Materials.

#### 3.2.3. Synthesis of 4-Hydroxymethyl-2,3-diphenylisoxazolidine (**13**–**18**)

Compounds **1** or **2**–**6** (50 mg, 0.144 mmol) and NaBH_4_ (21.79 mg, 0.576 mmol) were added to THF (4 mL) at 0 °C under a nitrogen atmosphere. The mixture was gradually returned to room temperature and continuously stirred for 5 h, and the reaction was monitored by TLC (R_f_ = 0.33 (petroleum ether/ethyl acetate = 2/1)). The reaction mixture was quenched with a small amount of water, and then washed with saturated salt water and extracted with ethyl acetate, and the organic phase was evaporated in a vacuum. The crude product was purified by preparative TLC (eluent: PE/EA = 3/1) to obtain products **13**–**18**. Corresponding data and spectra are given in the Supplementary Materials.

#### 3.2.4. Synthesis of 2-(2-(Aryl)-3-arylisoxazolidin-4-yl)propan-2-ol (**19**–**24**)

Compounds **1** or **2**–**6** (50 mg, 0.144 mmol) were dissolved in THF (4 mL), and next, a 3.0 M CH_3_MgBr solution in Et_2_O (480 μL, 1.44 mmol) was added dropwise at 0 °C under a nitrogen atmosphere. The mixture was gradually returned to room temperature and continuously stirred for 2 h, and the reaction was monitored by TLC (R_f_ = 0.3 (PE/EA = 5/1)). The reaction mixture was quenched with a small amount of water, and then washed with saturated salt water and extracted with ethyl acetate, and the organic phase was evaporated in a vacuum. The crude product was purified by preparative TLC (eluent: PE/EA = 4/1) to obtain products **19**–**24**. Corresponding data and spectra are given in the Supplementary Materials.

#### 3.2.5. Synthesis of 4-Acetyl-2,3-diarylisoxazolidines (**25**–**28**)

Compounds **1** or **2**–**6** (50 mg, 0.144 mmol) and Fe powder (25 mg, 0.447 mmol) were dissolved in CH_2_Cl_2_ (4 mL), and next, a 0.5 M (CH_3_)_2_CuLi solution in Et_2_O (864 μL, 0.432 mmol) was added dropwise at 0 °C under a nitrogen atmosphere. The mixture was gradually returned to room temperature and continuously stirred for 1 h, and the reaction was monitored by TLC (R_f_ = 0.6 (PE/EA = 5/1)). The reaction mixture was quenched with a small amount of water, and then washed with saturated salt water and extracted with ethyl acetate, and the organic phase was evaporated in a vacuum. The crude product was purified by preparative TLC (eluent: PE/EA = 10/1) to gain products **25**–**28**. Corresponding data and spectra are given in the Supplementary Materials.

#### 3.2.6. Synthesis of 2,3-Diaryl-4-ylcarbonylhydrazineisoxazolidines (**29**–**34**)

Compounds **1** or **2**–**6** (50 mg, 0.144 mmol) were dissolved in CH_2_Cl_2_ (4 mL), and next, 80*w*% hydrazine hydrate (1 mL, 19.2 mmol) was added dropwise at 0 °C. The mixture was gradually returned to room temperature and continuously stirred for 3 h, and the reaction was monitored by TLC (R_f_ = 0.1 (PE/EA = 1/1)). The reaction mixture was washed with saturated salt water and extracted with CH_2_Cl_2_, and the organic phase was evaporated in a vacuum. The crude product was crystallized from a mixture of PE-EA (10:1, *v*/*v*) to furnish products **29**–**34**. Corresponding data and spectra are given in the Supplementary Materials.

#### 3.2.7. Synthesis of Ethyl 2,3-Diarylisoxazolidin-4-ylcarboxylate (**35**–**40**)

Compounds **1** or **2**–**6** (50 mg, 0.144 mmol) were dissolved in CH_2_Cl_2_ (4 mL), and next, a solution of EtONa (10.75 mg, 0.158 mmol) in ethyl alcohol (1 mL) was added dropwise at 0 °C. The mixture was stirred for 5 min and the reaction was monitored by TLC (R_f_ = 0.8 (PE/EA = 10/1)). The reaction mixture was quenched with a small amount of water, the solvent was evaporated in vacuum, and the mixture was then washed with saturated salt water, extracted with ethyl acetate, and the organic phase was evaporated in vacuum. The crude product was then purified by preparative TLC (eluent: PE/EA = 20/1) to build products **35**–**40**. Corresponding data and spectra are given in the Supplementary Materials.

### 3.3. X-ray Structural Analysis of Compound ***6***

A crystal of compound **6** was gained by recrystallization from the CH_2_Cl_2_ solution. X-ray single-crystal structural data were obtained with a Bruker Apex Smart CCDC Venture diffractometer at 293(2)K. CCDC 2234690 contains the supplementary crystallographic data for compound **6**. These data are accessed on 6 January 2023 and can be obtained free of charge via http://www.ccdc.cam.ac.uk/conts/retrieving.html (or from the CCDC, 12 Union Road, Cambridge CB2 1EZ, UK; Fax: +44-1223-336033; E-mail: deposit@ccdc.cam.ac.uk).

## 4. Conclusions

In summary, 4-isoxazolidine intermediates featuring a 3,5-dimethylpyrazolylcarbonyl side group were selected as befitting substrates which might be used to construct novel diversified isoxazolidine derivatives by six kinds of transformations, like ring-opening, nucleophilic substitution, addition-elimination and reduction. The results showed that 5% Pd/C induced the ring-opening reaction of isoxazolidine, forming 2-(3,5-dimethylpyrazol-1-ylcarbonyl)-3-(arylamino)-3-arylpropan-1-ol, and there was no influence on the 3,5-dimethylpyrazolylcarbonyl side group. The isoxazolidine ring was selectively retained and the pyrazolylcarbonyl portion was successfully reduced to primary alcohol by the use of a NaBH_4_ reducing agent. Utilizing the good reactivity and leaving-group ability of the 3,5-dimethylpyrazolylcarbonyl side group, the 4-isoxazolidine derivatives containing varying functional groups (-CH_2_OH, -C(CH_3_)_2_OH, -COCH_3_, -COOC_2_H_5_, and -CONHNH_2_) were expediently produced in generally good-to-high yields (up to 95% yield) under mild conditions. The method described provides a valuable reference for the derivations of other heterocyclic compounds, and the construction of isoxazolidines featuring diverse functional groups may offer a more comprehensive structural database for the development of new drugs and functional materials.

## Figures and Tables

**Table 1:** Effect of nitrone substituents on 1,3-dipolar cycloaddition.

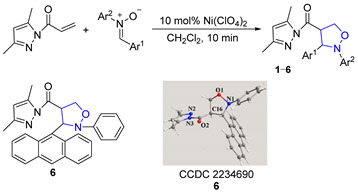				
Entry	Substrate		Product	Yield ^a^/%
	Ar^1^	Ar^2^		
1	Phenyl	Phenyl	**1**	90
2	Phenyl	4-CH_3_ Phenyl	**2**	99
3	Phenyl	4-C_2_H_5_ Phenyl	**3**	99
4	Phenyl	4-Cl Phenyl	**4**	80
5	Phenyl	3-CN Phenyl	**5**	85
6	9-anthryl	Phenyl	**6**	70

^a^ Isolated yields.

**Table 2:** Effects of different groups of (pyrazolylcarbonyl)isoxazolidines on the Pd/C reduction reaction.

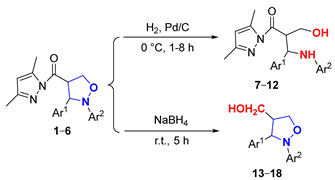					
Entry	Substrate		Time/h	Product	Yield ^a^/%
	Ar^1^	Ar^2^			
1	Phenyl	Phenyl	2	**7**	87
2	Phenyl	4-CH_3_ Phenyl	1	**8**	95
3	Phenyl	4-C_2_H_5_ Phenyl	1	**9**	95
4	Phenyl	4-Cl Phenyl	8	**10**	70
5	Phenyl	3-CN Phenyl	3	**11**	84
6	9-anthryl	Phenyl	8	**12**	60
7	Phenyl	Phenyl	5	**1** **3**	92
8	Phenyl	4-CH_3_ Phenyl	5	**1** **4**	94
9	Phenyl	4-C_2_H_5_ Phenyl	5	**1** **5**	93
10	Phenyl	4-Cl Phenyl	5	**1** **6**	92
11	Phenyl	3-CN Phenyl	5	**1** **7**	94
12	9-anthryl	Phenyl	5	**1** **8**	91

^a^ Isolated yields.

**Table 3:** Effect of different reaction factors on a metallic organics nucleophilic substitution reaction.

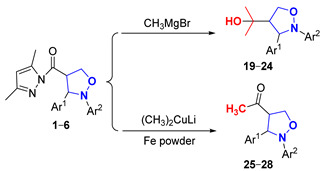								
Entry	Substrate		Reagent	Equiv. Ratio	Temp./°C	Time/h	Product	Yield ^a^/%
	Ar^1^	Ar^2^						
1	Phenyl	Phenyl	CH_3_MgBr	0.8	0	2	**19**	-
2	Phenyl	Phenyl	CH_3_MgBr	1.0	0	2	**19**	-
3	Phenyl	Phenyl	CH_3_MgBr	2.0	0	2	**19**	7
4	Phenyl	Phenyl	CH_3_MgBr	5.0	0	2	**19**	30
5	Phenyl	Phenyl	CH_3_MgBr	7.0	0	2	**19**	56
6	Phenyl	Phenyl	CH_3_MgBr	9.0	0	2	**19**	70
7	Phenyl	Phenyl	CH_3_MgBr	10.0	0	2	**19**	73
8	Phenyl	Phenyl	CH_3_MgBr	11.0	0	0.5	**19**	73
9	Phenyl	Phenyl	CH_3_MgBr	11.0	r.t.	0.5	**19**	Trace
10	Phenyl	Phenyl	CH_3_MgBr	11.0	−10	3	**19**	65
11	Phenyl	Phenyl	CH_3_MgBr	11.0	−20	3	**19**	52
12	Phenyl	4-CH_3_ Phenyl	CH_3_MgBr	10.0	0	2	**20**	75
13	Phenyl	4-C_2_H_5_ Phenyl	CH_3_MgBr	10.0	0	2	**21**	75
14	Phenyl	4-Cl Phenyl	CH_3_MgBr	10.0	0	2	**22**	72
15	Phenyl	3-CN Phenyl	CH_3_MgBr	10.0	0	2	**23**	73
16	9-anthryl	Phenyl	CH_3_MgBr	10.0	0	2	**24**	70
17	Phenyl	Phenyl	(CH_3_)_2_CuLi	3.0	0	1	**25**	-
18	Phenyl	Phenyl	(CH_3_)_2_CuLi/Fe	3.0	0	1	**25**	69
19	Phenyl	4-CH_3_ Phenyl	(CH_3_)_2_CuLi/Fe	3.0	0	1	**26**	70
20	Phenyl	4-C_2_H_5_ Phenyl	(CH_3_)_2_CuLi/Fe	3.0	0	1	**27**	70
21	Phenyl	4-Cl Phenyl	(CH_3_)_2_CuLi/Fe	3.0	0	1	**28**	68

^a^ Isolated yields.

**Table 4:** Effect of (pyrazolylcarbonyl)isoxazolidine substituents on hydrazinolysis reactions.

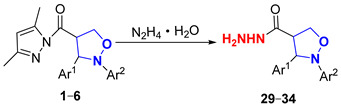						
Entry	Substrate		Temp./°C	Time/h	Product	Yield ^a^/%
	Ar^1^	Ar^2^				
1	Phenyl	Phenyl	0	1	**29**	70
2	Phenyl	Phenyl	r.t.	1	**29**	45
3	Phenyl	Phenyl	0	2	**29**	85
4	Phenyl	Phenyl	0	3	**29**	92
5	Phenyl	Phenyl	0	4	**29**	93
6	Phenyl	4-CH_3_ Phenyl	0	3	**3** **0**	93
7	Phenyl	4-C_2_H_5_ Phenyl	0	3	**3** **1**	95
8	Phenyl	4-Cl Phenyl	0	3	**3** **2**	92
9	Phenyl	3-CN Phenyl	0	3	**3** **3**	91
10	9-anthryl	Phenyl	0	3	**3** **4**	90

^a^ Isolated yields.

**Table 5:** Effect of different reaction factors on the EtONa alcoholysis reaction.

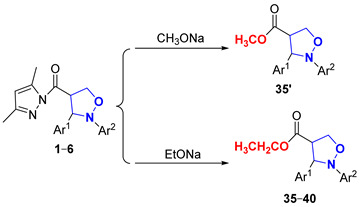								
Entry	Substrate		Reagent	Equiv. Ratio	Temp./°C	Time/min	Product	Yield ^a^/%
	Ar^1^	Ar^2^						
1	Phenyl	Phenyl	CH_3_ONa	0.8	r.t.	30	-	-
2	Phenyl	Phenyl	CH_3_ONa	0.8	0	5	**3** **5′**	60
3	Phenyl	Phenyl	CH_3_ONa	1.1	0	5	**3** **5′**	73
4	Phenyl	Phenyl	EtONa	1.1	0	5	**3** **5**	93
5	Phenyl	Phenyl	EtONa	1.1	0	10	**3** **5**	53
6	Phenyl	Phenyl	EtONa	1.1	0	15	**3** **5**	37
7	Phenyl	Phenyl	EtONa	1.1	0	20	**3** **5**	14
8	Phenyl	Phenyl	EtONa	1.1	0	25	**3** **5**	Trace
9	Phenyl	Phenyl	EtONa	0.8	0	5	**3** **5**	41
10	Phenyl	Phenyl	EtONa	0.5	0	5	**3** **5**	22
11	Phenyl	4-CH_3_ Phenyl	EtONa	1.1	0	5	**3** **6**	93
12	Phenyl	4-C_2_H_5_ Phenyl	EtONa	1.1	0	5	**3** **7**	93
13	Phenyl	4-Cl Phenyl	EtONa	1.1	0	5	**38**	92
14	Phenyl	3-CN Phenyl	EtONa	1.1	0	5	**39**	94
15	9-anthryl	Phenyl	EtONa	1.1	0	5	**4** **0**	93

^a^ Isolated yields.

## Data Availability

The original contributions presented in the study are included in the Article/Supplementary Materials; further inquiries can be directed to the corresponding authors.

## Supplementary Materials

The following supporting information can be downloaded at: https://www.mdpi.com/article/10.3390/molecules29153454/s1. Spectral data and references are available. Figures S1–S40: ^1^H and ^13^C NMR spectra; Tables S1 and S2: Crystallographic data of compound **6**.
