# Supplementary material for: Synthesis of New Isoxazolidine Derivatives Utilizing the Functionality of N-Carbonylpyrazol-Linked Isoxazolidines
Source: Molecules. 2024 Jul 23;29(15):3454. doi: 10.3390/molecules29153454 (PMC11314590; doi:10.3390/molecules29153454)
Supplement: Supplementary file 1 [file molecules-29-03454-s001.zip › molecules_cmpd-check-list.pdf]

molecules

MDPI

Chemical Compounds Characterization Checklist

Manuscript ID: ______________________

Submitting Author: Xixian Cao, Jun You *, Yunze Wang, Yanchao Yu, Wenju Wu and

Compound			Characterization											
Compound Nr.	New	Known (Ref. in main text)	Physical State (purity %)	1H NMR	13C NMR	2D NMR	HRMS	IR	XRD	GC/ HPLC				Data in SI
1	☐	[8]	solid	☑	☑	☐	☐	☑	☐	☐	☐	☐	☐	☑
2	☑		solid	☑	☑	☐	☑	☑	☐	☐	☐	☐	☐	☑
3	☑		solid	☑	☑	☐	☑	☑	☐	☐	☐	☐	☐	☑
4	☑		solid	☑	☑	☐	☑	☑	☐	☐	☐	☐	☐	☑
5	☑		solid	☑	☑	☐	☑	☑	☐	☐	☐	☐	☐	☑
6	☑		solid	☑	☑	☐	☑	☑	☑	☐	☐	☐	☐	☑
7	☑		oily liquid	☑	☑	☐	☑	☑	☐	☐	☐	☐	☐	☑
8	☑		oily liquid	☑	☑	☐	☑	☑	☐	☐	☐	☐	☐	☑
9	☑		oily liquid	☑	☑	☐	☑	☑	☐	☐	☐	☐	☐	☑
10	☑		oily liquid	☑	☑	☐	☑	☑	☐	☐	☐	☐	☐	☑
11	☑		oily liquid	☑	☑	☐	☑	☑	☐	☐	☐	☐	☐	☑
12	☑		oily liquid	☑	☑	☐	☑	☑	☐	☐	☐	☐	☐	☑

Note: insert the relevant information and select only the techniques used in this study. In the empty columns you can insert any additional methods.

molecules

MDPI

Compound			Characterization											
Compound Nr.	New	Known (Ref. in main text)	Physical State (purity %)	1H NMR	13C NMR	2D NMR	HRMS	IR	XRD	GC/ HPLC				Data in SI
13	☑		oily liquid	☑	☑	☐	☑	☑	☐	☐	☐	☐	☐	☑
14	☑		oily liquid	☑	☑	☐	☑	☑	☐	☐	☐	☐	☐	☑
15	☑		oily liquid	☑	☑	☐	☑	☑	☐	☐	☐	☐	☐	☑
16	☑		oily liquid	☑	☑	☐	☑	☑	☐	☐	☐	☐	☐	☑
17	☑		oily liquid	☑	☑	☐	☑	☑	☐	☐	☐	☐	☐	☑
18	☑		oily liquid	☑	☑	☐	☑	☑	☐	☐	☐	☐	☐	☑
19	☑		oily liquid	☑	☑	☐	☑	☑	☐	☐	☐	☐	☐	☑
20	☑		oily liquid	☑	☑	☐	☑	☑	☐	☐	☐	☐	☐	☑
21	☑		oily liquid	☑	☑	☐	☑	☑	☐	☐	☐	☐	☐	☑
22	☑		oily liquid	☑	☑	☐	☑	☑	☐	☐	☐	☐	☐	☑
23	☑		oily liquid	☑	☑	☐	☑	☑	☐	☐	☐	☐	☐	☑
24	☑		oily liquid	☑	☑	☐	☑	☑	☐	☐	☐	☐	☐	☑
25	☑		oily liquid	☑	☑	☐	☑	☑	☐	☐	☐	☐	☐	☑
26	☑		oily liquid	☑	☑	☐	☑	☑	☐	☐	☐	☐	☐	☑
27	☑		oily liquid	☑	☑	☐	☑	☑	☐	☐	☐	☐	☐	☑
28	☑		oily liquid	☑	☑	☐	☑	☑	☐	☐	☐	☐	☐	☑

molecules

MDPI

Compound			Characterization											
Compound Nr.	New	Known (Ref. in main text)	Physical State (purity %)	1H NMR	13C NMR	2D NMR	HRMS	IR	XRD	GC/ HPLC				Data in SI
29	☑		solid	☑	☑	☐	☑	☑	☐	☐	☐	☐	☐	☑
30	☑		solid	☑	☑	☐	☑	☑	☐	☐	☐	☐	☐	☑
31	☑		solid	☑	☑	☐	☑	☑	☐	☐	☐	☐	☐	☑
32	☑		solid	☑	☑	☐	☑	☑	☐	☐	☐	☐	☐	☑
33	☑		solid	☑	☑	☐	☑	☑	☐	☐	☐	☐	☐	☑
34	☑		solid	☑	☑	☐	☑	☑	☐	☐	☐	☐	☐	☑
35	☑		oily liquid	☑	☑	☐	☑	☑	☐	☐	☐	☐	☐	☑
36	☑		oily liquid	☑	☑	☐	☑	☑	☐	☐	☐	☐	☐	☑
37	☑		oily liquid	☑	☑	☐	☑	☑	☐	☐	☐	☐	☐	☑
38	☑		oily liquid	☑	☑	☐	☑	☑	☐	☐	☐	☐	☐	☑
39	☑		oily liquid	☑	☑	☐	☑	☑	☐	☐	☐	☐	☐	☑
40	☑		oily liquid	☑	☑	☐	☑	☑	☐	☐	☐	☐	☐	☑
	☐			☐	☐	☐	☐	☐	☐	☐	☐	☐	☐	☐
	☐			☐	☐	☐	☐	☐	☐	☐	☐	☐	☐	☐
	☐			☐	☐	☐	☐	☐	☐	☐	☐	☐	☐	☐
	☐			☐	☐	☐	☐	☐	☐	☐	☐	☐	☐	☐

Compound			Characterization											
Compound Nr.	New	Known (Ref. in main text)	Physical State (purity %)	1H NMR	13C NMR	2D NMR	HRMS	IR	XRD	GC/ HPLC				Data in SI
	☐			☐	☐	☐	☐	☐	☐	☐	☐	☐	☐	☐
	☐			☐	☐	☐	☐	☐	☐	☐	☐	☐	☐	☐
	☐			☐	☐	☐	☐	☐	☐	☐	☐	☐	☐	☐
	☐			☐	☐	☐	☐	☐	☐	☐	☐	☐	☐	☐
	☐			☐	☐	☐	☐	☐	☐	☐	☐	☐	☐	☐
	☐			☐	☐	☐	☐	☐	☐	☐	☐	☐	☐	☐
	☐			☐	☐	☐	☐	☐	☐	☐	☐	☐	☐	☐
	☐			☐	☐	☐	☐	☐	☐	☐	☐	☐	☐	☐
	☐			☐	☐	☐	☐	☐	☐	☐	☐	☐	☐	☐
	☐			☐	☐	☐	☐	☐	☐	☐	☐	☐	☐	☐
	☐			☐	☐	☐	☐	☐	☐	☐	☐	☐	☐	☐
	☐			☐	☐	☐	☐	☐	☐	☐	☐	☐	☐	☐
	☐			☐	☐	☐	☐	☐	☐	☐	☐	☐	☐	☐
	☐			☐	☐	☐	☐	☐	☐	☐	☐	☐	☐	☐
	☐			☐	☐	☐	☐	☐	☐	☐	☐	☐	☐	☐
	☐			☐	☐	☐	☐	☐	☐	☐	☐	☐	☐	☐

Compound			Characterization											
Compound Nr.	New	Known (Ref. in main text)	Physical State (purity %)	1H NMR	13C NMR	2D NMR	HRMS	IR	XRD	GC/ HPLC				Data in SI
	☐			☐	☐	☐	☐	☐	☐	☐	☐	☐	☐	☐
	☐			☐	☐	☐	☐	☐	☐	☐	☐	☐	☐	☐
	☐			☐	☐	☐	☐	☐	☐	☐	☐	☐	☐	☐
	☐			☐	☐	☐	☐	☐	☐	☐	☐	☐	☐	☐
	☐			☐	☐	☐	☐	☐	☐	☐	☐	☐	☐	☐
	☐			☐	☐	☐	☐	☐	☐	☐	☐	☐	☐	☐
	☐			☐	☐	☐	☐	☐	☐	☐	☐	☐	☐	☐
	☐			☐	☐	☐	☐	☐	☐	☐	☐	☐	☐	☐
	☐			☐	☐	☐	☐	☐	☐	☐	☐	☐	☐	☐
	☐			☐	☐	☐	☐	☐	☐	☐	☐	☐	☐	☐
	☐			☐	☐	☐	☐	☐	☐	☐	☐	☐	☐	☐
	☐			☐	☐	☐	☐	☐	☐	☐	☐	☐	☐	☐
	☐			☐	☐	☐	☐	☐	☐	☐	☐	☐	☐	☐
	☐			☐	☐	☐	☐	☐	☐	☐	☐	☐	☐	☐
	☐			☐	☐	☐	☐	☐	☐	☐	☐	☐	☐	☐
	☐			☐	☐	☐	☐	☐	☐	☐	☐	☐	☐	☐
