# Supplementary material for: Synthesis of New Isoxazolidine Derivatives Utilizing the Functionality of N-Carbonylpyrazol-Linked Isoxazolidines
Source: Molecules. 2024 Jul 23;29(15):3454. doi: 10.3390/molecules29153454 (PMC11314590; doi:10.3390/molecules29153454)
Supplement: Supplementary file 1 [file molecules-29-03454-s001.zip › Revised Supporting information (with changes marked).pdf]

Supporting Information

Synthesis of New Isoxazolidine Derivatives Utilizing the Functionality of *N*-Carbonylpyrazol-Linked Isoxazolidines

Xixian Cao, Jun You *, Yunze Wang, Yanchao Yu, Wenju Wu and Yifang Liang *

Key Laboratory of Green Chemical Engineering and Technology of Heilongjiang Province, College of Materials Science and Chemical Engineering, Harbin University of Science and Technology, Harbin 150080, China

* Correspondence: youjunjun@126.com (J.Y.); liangyifang960701@163.com (Y.L.)

Contents

1. Spectral data of compounds **1-40**. 1
2. ^{1}H and ^{13}C NMR spectra of compounds **1-40**........ 21
3. Crystallographic data of compound **6**........ 36
4. Reference........ 38

1. Spectral data of compounds **1-40**.

4-(3,5-Dimethylpyrazol-1-ylcarbonyl)-2,3-diphenylisoxazolidine (**1**) [1]: Yield 0.93 g (90%), yellowish solid. IR spectrum, ν, cm^{-1}: 3031, 2927, 2874, 1723, 1597, 1585, 1488,

1382, 1337, 1257, 1027, 963, 817, 752, 696. ^{1}H NMR spectrum (300 MHz), δ, ppm (*J*, Hz): 2.21 (3H, s, CH_3); 2.51 (3H, s, CH_3); 4.23−4.28 (1H, m, H-4); 4.71−4.78 (2H, m, H-5); 5.37 (1H, d, *J* = 5.2, H-3); 5.97 (1H, s, H-4 pyrazole); 6.96 (1H, ddt, *J* = 7.3, *J* = 5.8, *J* = 1.1, H Ph); 7.00−7.08 (2H, m, H Ph); 7.23−7.34 (3H, m, H Ph); 7.37−7.42 (2H, m, H Ph); 7.61−7.63 (2H, m, H Ph). ^{13}C NMR spectrum (75 MHz), δ, ppm: 13.8, 14.3 (C-3 pyrazole-$\underline{C}H_3$ and C-5 pyrazole-$\underline{C}H_3$); 59.7 (C-4); 70.1 (C-5); 70.7 (C-3); 111.5 (C-4 pyrazole); 115.2, 126.6, 126.9 (2-(C-2−6 Ph)); 127.6, 128.8, 128.9 (3-(C-2−6 Ph)); 141.5 (3-(C-1 Ph)); 144.5 (C-5 pyrazole); 150.5 (2-(C-1 Ph)); 152.6 (C-3 pyrazole); 170.5 (C=O).

4-(3,5-Dimethylpyrazol-1-ylcarbonyl)-2-(4-methylphenyl)-3-phenylisoxazolidine (**2**): Yield 1.06 g (99%), yellowish solid, mp 115.4–116.0 °C. IR spectrum, ν, cm^{-1}: 3028, 2924, 2847, 1724, 1611, 1585, 1507, 1454, 1382, 1337, 1255, 1027, 963, 813, 762, 700. ^{1}H NMR spectrum (300 MHz), δ, ppm (*J*, Hz): 2.19 (3H, s, CH_3); 2.28 (3H, s, 2-(4 Ph-CH_3)); 2.51 (3H, s, CH_3); 4.21−4.26 (1H, m, H-4); 4.65−4.78 (2H, m, H-5); 5.26 (1H, d, *J* = 6.5, H-3); 5.96 (1H, s, H-4 pyrazole); 6.97 (2H, d, *J* = 9.5, H Ph); 7.06 (2H, d, *J* = 9.2, H Ph); 7.32−7.40 (3H, m, H Ph); 7.59 (2H, d, *J* = 6.3, H Ph). ^{13}C NMR spectrum (75 MHz), δ, ppm: 13.8, 14.3 (C-3 pyrazole-$\underline{C}H_3$ and C-5 pyrazole-$\underline{C}H_3$); 20.6 (2-(C-4 Ph-$\underline{C}H_3$)); 59.5 (C-4); 70.0 (C-5); 70.9 (C-3); 111.4 (C-4 pyrazole); 115.8, 127.1, 127.5 (2-(C-2−6 Ph)); 128.5, 128.7, 129.3 (3-(C-2−6 Ph)); 141.3 (3-(C-1 Ph)); 144.4 (C-5 pyrazole); 148.0 (2-(C-1 Ph)); 152.5 (C-3 pyrazole); 170.7 (C=O). Found, *m*/*z*: 360.1750 [M–H]$^-$. $C_{22}H_{22}N_3O_2$ Calculated, *m*/*z*: 360.1790.

4-(3,5-Dimethylpyrazol-1-ylcarbonyl)-2-(4-ethylphenyl)-3-phenylisoxazolidine (**3**): Yield 1.11 g (99%), yellowish solid, mp 122.7–123.3 °C. IR spectrum, ν, cm^{-1}: 2962, 2927,

2871, 1724, 1610, 1585, 1507, 1382, 1339, 1255, 1027, 963, 831, 700. ^{1}H NMR spectrum (300 MHz), δ, ppm (*J*, Hz): 1.20 (3H, t, *J* = 6.3, 2-(4 Ph-$CH_2C\underline{H}_3$)); 2.20 (3H, s, CH_3); 2.51 (3H, s, CH_3); 2.54–2.62 (2H, m, 2-(4 Ph-$C\underline{H}_2CH_3$)); 4.22–4.27 (1H, m, H-4); 4.66–4.77 (2H, m, H-5); 5.28 (1H, d, *J* = 6.4, H-3); 5.96 (1H, s, H-4 pyrazole); 6.99 (2H, d, *J* = 9.5, H Ph); 7.08 (2H, d, *J* = 9.3, H Ph); 7.30–7.62 (5H, m, H Ph). ^{13}C NMR spectrum (75 MHz), δ, ppm: 13.6, 13.8 (C-3 pyrazole-$\underline{C}H_3$ and C-5 pyrazole-$\underline{C}H_3$); 14.3 (2-(C-4 Ph-$CH_2\underline{C}H_3$)); 28.0 (2-(C-4 Ph-$\underline{C}H_2CH_3$)); 59.6 (C-4); 70.0 (C-5); 70.8 (C-3); 111.4 (C-4 pyrazole); 115.8, 127.0, 127.5 (2-(C-2−6 Ph)); 128.1, 128.7, 138.1 (3-(C-2−6 Ph)); 141.4 (3-(C-1 Ph)); 144.4 (C-5 pyrazole); 148.2 (2-(C-1 Ph)); 152.5 (C-3 pyrazole); 170.6 (C=O). Found, *m*/*z*: 374.2230 $[M–H]^-$. $C_{23}H_{24}N_3O_2$ Calculated, *m*/*z*: 374.2247.

4-(3,5-Dimethylpyrazol-1-ylcarbonyl)-2-(4-chlorophenyl)-3-phenylisoxazolidine (**4**): Yield 0.91 g (80%), yellowish solid, mp 124.2–124.8 °C. IR spectrum, ν, cm^{-1}: 2945, 1723, 1586, 1487, 1382, 1338, 1256, 962, 824, 751, 700. ^{1}H NMR spectrum (300 MHz), δ, ppm (*J*, Hz): 2.20 (3H, s, CH_3); 2.51 (3H, s, CH_3); 4.21–4.25 (1H, m, H-4); 4.68–4.79 (2H, m, H-5); 5.27 (1H, d, *J* = 6.1, H-3); 5.97 (1H, s, H-4 pyrazole); 6.98 (2H, d, *J* = 9.5, H Ph); 7.20 (2H, d, *J* = 9.0, H Ph);7.31–7.41 (3H, m, H Ph); 7.58 (2H, d, *J* = 9.2, H Ph). ^{13}C NMR spectrum (75 MHz), δ, ppm: 13.8, 14.3 (C-3 pyrazole-$\underline{C}H_3$ and C-5 pyrazole-$\underline{C}H_3$); 59.5 (C-4); 70.2 (C-5); 70.7 (C-3); 111.5 (C-4 pyrazole); 116.6, 126.6, 126.9 (2-(C-2−6 Ph)); 128.7, 128.8, 128.9 (3-(C-2−6 Ph)); 140.9 (3-(C-1 Ph)); 144.5 (C-5 pyrazole); 149.1 (2-(C-1 Ph)); 152.7 (C-3 pyrazole); 170.4 (C=O). Found, *m*/*z*: 380.1181 $[M–H]^-$. $C_{21}H_{19}ClN_3O_2$ Calculated, *m*/*z*: 380.1244.

4-(3,5-Dimethylpyrazol-1-ylcarbonyl)-2-(3-cyanophenyl)-3-phenylisoxazolidine (**5**):

Yield 0.94 g (85%), yellowish solid, mp 130.1–130.7 °C. IR spectrum, ν, cm^{-1}: 2926, 2229, 1723, 1597, 1479, 1383, 1339, 1258, 1027, 962, 789, 701. ^{1}H NMR spectrum (300 MHz), δ, ppm (*J*, Hz): 2.20 (3H, s, CH_3); 2.51 (3H, s, CH_3); 4.20–4.24 (1H, m, H-4); 4.71–4.80 (2H, m, H-5); 5.33 (1H, d, *J* = 3.2, H-3); 5.98 (1H, s, H-4 pyrazole); 7.15–7.23 (2H, m, H Ph); 7.32–7.43 (5H, m, H Ph); 7.57 (2H, d, *J* = 9.7, H Ph). ^{13}C NMR spectrum (75 MHz), δ, ppm: 13.8, 14.3 (C-3 pyrazole-$\underline{C}H_3$ and C-5 pyrazole-$\underline{C}H_3$); 59.5 (C-4); 70.3 (C-5); 70.4 (C-3); 111.7 (C-4 pyrazole); 112.6, 117.8 (3-(C-2−6 Ph)); 119.0 (2-(C-3 Ph-$\underline{C}$N)); 126.7, 128.0, 128.5, 129.6 (2-(C-2, 4−6 Ph)); 130.1 (3-(C-1 Ph)); 133.6 (C-5 pyrazole); 140.6 (C-3 pyrazole); 144.6 (2-(C-1 Ph)); 170.1 (C=O). Found, *m*/*z*: 371.1548 $[M–H]^{-}$. $C_{22}H_{19}N_4O_2$ Calculated, *m*/*z*: 371.1586.

3-(9-Anthryl)-4-(3,5-dimethylpyrazol-1-ylcarbonyl)-2-phenylisoxazolidine (**6**): Yield 0.93 g (70%), yellowish solid, mp 149.9–150.5 °C. IR spectrum, ν, cm^{-1}: 3409, 3052, 2923, 1720, 1596, 1486, 1379, 1338, 1307, 1252, 1086, 1026, 962, 731. ^{1}H NMR spectrum (300 MHz), δ, ppm (*J*, Hz): 2.00 (3H, s, CH_3); 2.43 (3H, s, CH_3); 4.41–4.46 (1H, m, H-4); 5.14–5.52 (2H, m, H-5); 5.79 (1H, d, *J* = 8.3, H-3); 6.72 (1H, s, H-4 pyrazole); 6.92 (3H, d, *J* = 8.7, H Ph); 6.98 (2H, d, *J* = 7.2, H Ph); 8.06 (2H, d, *J* = 5.0, H Anthryl); 7.13–7.50 (4H, m, H Anthryl); 8.48 (1H, s, H Anthryl); 8.72 (1H, s, H Anthryl); 9.33 (1H, s, H Anthryl). ^{13}C NMR spectrum (75 MHz), δ, ppm: 13.5, 14.3 (C-3 pyrazole-$\underline{C}H_3$ and C-5 pyrazole-$\underline{C}H_3$); 57.7 (C-4); 68.5 (C-5); 71.9 (C-3); 111.4 (C-4 pyrazole); 111.5 (2-(C-2, 6 Ph)); 115.3, 122.1 (3-(C-3, 8, 13 Anthryl)); 128.8, 129.1 (2-(C-3−5 Ph)); 129.3, 129.4, 129.9, 130.1, 133.6, 140.9 (3-(C Anthryl)); 144.5 (C-5 pyrazole); 152.2 (2-(C-1 Ph)); 152.5 (C-3 pyrazole); 170.3 (C=O). Found, *m*/*z*: 448.2028 $[M+H]^{+}$. $C_{29}H_{26}N_3O_2$ Calculated, *m*/*z*: 448.1947.

2-(3,5-Dimethylpyrazol-1-ylcarbonyl)-3-(phenylamino)-3-phenylpropan-1-ol (**7**): Yield 43.75 mg (87%), colorless oily liquid. IR spectrum, ν, cm^{-1}: 3384, 3055, 3026, 2926, 1718, 1602, 1499, 1383, 1324, 1030, 951, 750, 700. ^1H NMR spectrum (300 MHz), δ, ppm (*J*, Hz): 2.34 (3H, s, CH_3); 2.47 (3H, s, CH_3); 2.66–2.69 (1H, m, H-2); 3.99–4.05 (2H, m, H-1); 4.35 (1H, td, *J* = 4.9, *J* = 3.5, H-3); 5.35 (1H, d, *J* = 4.8, H-4 pyrazole); 5.99 (1H, br. s, OH); 6.53 (2H, d, *J* = 6.3, H Ph); 6.64–6.68 (1H, m, H Ph); 7.07 (2H, d, *J* = 6.2, H Ph); 7.25–7.37 (3H, m, H Ph); 7.47 (2H, d, *J* = 6.0, H Ph); 8.04 (1H, br. s, NH). ^{13}C NMR spectrum (75 MHz), δ, ppm: 14.2, 14.4 (C-3 pyrazole-$\underline{C}H_3$ and C-5 pyrazole-$\underline{C}H_3$); 51.3 (C-2); 58.4 (C-3); 60.8 (C-1); 111.6 (C-4 pyrazole); 113.8, 126.8, 127.3 (NH-(C-2−6 Ph)); 128.5, 128.6, 129.0 (3-(C-2−6 Ph)); 140.4 (3-(C-1 Ph)); 144.4 (C-5 pyrazole); 146.7 (NH-(C-1 Ph)); 152.7 (C-3 pyrazole); 174.0 (C=O). Found, *m*/*z*: 348.1750 $[M–H]^-$. $C_{21}H_{22}N_3O_2$ Calculated, *m*/*z*: 348.1790.

2-(3,5-Dimethylpyrazol-1-ylcarbonyl)-3-(4-methylphenylamino)-3-phenylpropan-1-ol (**8**): 47.76 mg Yield (95%), colorless oily liquid. IR spectrum, ν, cm^{-1}: 3378, 3026, 2925, 1719, 1617, 1585, 1520, 1382, 1325, 1269, 1040, 951, 909, 807, 733, 701. ^1H NMR spectrum (300 MHz), δ, ppm (*J*, Hz): 2.19 (3H, s, CH_3); 2.33 (3H, s, 2-(4 Ph-C$\underline{H}_3$)); 2.46 (3H, s, CH_3); 2.68–2.70 (1H, m, H-2); 3.93–4.07 (2H, m, H-1); 4.35 (1H, td, *J* = 9.0, *J* = 3.5, H-3); 5.30 (1H, d, *J* = 3.0, H-4 pyrazole); 5.98 (1H, br. s, OH); 6.44 (2H, d, *J* = 6.5, H Ph); 6.89 (2H, d, *J* = 9.2, H Ph); 7.24–7.35 (3H, m, H Ph); 7.45 (2H, d, *J* = 6.2, H Ph); 8.03 (1H, br. s, NH). ^{13}C NMR spectrum (75 MHz), δ, ppm: 14.0, 14.4 (C-3 pyrazole-$\underline{C}H_3$ and C-5 pyrazole-$\underline{C}H_3$); 20.3 (NH-(C-4 Ph-$\underline{C}H_3$)); 51.4 (C-2); 58.6 (C-3); 60.8 (C-1); 111.5 (C-4 pyrazole); 113.9, 126.8, 126.8 (NH-(C-2−6 Ph)); 127.2, 128.5, 129.5 (3-(C-2−6 Ph));

140.6 (3-(C-1 Ph)); 144.5 (NH-(C-1 Ph)); 148.0 (C-5 pyrazole); 152.6 (C-3 pyrazole); 174.1 (C=O). Found, *m*/*z*: 362.1902 [M–H]$^-$. $C_{22}H_{24}N_3O_2$ Calculated, *m*/*z*: 362.1947.

2-(3,5-Dimethylpyrazol-1-ylcarbonyl)-3-(4-ethylphenylamino)-3-phenylpropan-1-ol (**9**): Yield 47.75 mg (95%), colorless oily liquid. IR spectrum, ν, cm^{-1}: 3386, 3026, 2961, 2927, 1719, 1616, 1585, 1519, 1382, 1325, 1262, 1030, 952, 909, 820, 732, 701. ^{1}H NMR spectrum (300 MHz), δ, ppm (*J*, Hz): 1.15 (3H, t, *J* = 9.1, 2-(4 Ph-$CH_2C\underline{H}_3$)); 2.34 (3H, s, CH_3); 2.28 (3H, s, CH_3); 2.47–2.57 (2H, m, 2-(4 Ph-$C\underline{H}_2CH_3$)); 4.00–4.03 (2H, m, H-1); 4.04–4.07 (1H, m, H-2); 4.37 (1H, td, *J* = 5.1, *J* = 3.6, H-3); 5.32 (1H, d, *J* = 4.9, H-4 pyrazole); 5.99 (1H, br. s, OH); 6.48 (2H, d, *J* = 9.3, H Ph); 6.94 (2H, d, *J* = 9.5, H Ph); 7.23–7.37 (3H, m, H Ph); 7.48 (2H, d, *J* = 6.2, H Ph); 8.11 (1H, br. s, NH). ^{13}C NMR spectrum (75 MHz), δ, ppm: 14.0, 14.5 (C-3 pyrazole-$\underline{C}H_3$ and C-5 pyrazole-$\underline{C}H_3$); 15.9 ((NH-(C-4 Ph-$CH_2\underline{C}H_3$)); 27.8 (NH-(C-4 Ph-$\underline{C}H_2CH_3$)); 51.5 (C-2); 58.6 (C-3); 60.8 (C-1); 111.6 (C-4 pyrazole); 113.9, 126.8, 127.3 (NH-(C-2−6 Ph)); 128.3, 128.5, 133.3 (3-(C-2−6 Ph)); 140.8 (3-(C-1 Ph)); 144.4 (C-5 pyrazole); 144.8 (NH-(C-1 Ph)); 152.6 (C-3 pyrazole); 174.0 (C=O). Found, *m*/*z*: 376.2065 [M–H]$^-$. $C_{23}H_{26}N_3O_2$ Calculated, *m*/*z*: 376.2103.

2-(3,5-Dimethylpyrazol-1-ylcarbonyl)-3-(4-chlorophenylamino)-3-phenylpropan-1-ol (**10**): Yield 35.18 mg (70%), colorless oily liquid. IR spectrum, ν, cm^{-1}: 3377, 2959, 2924, 2850, 1715, 1599, 1495, 1454, 1382, 1325, 1259, 1176, 1091, 1043, 952, 815, 701. ^{1}H NMR spectrum (300 MHz), δ, ppm (*J*, Hz): 2.33 (3H, s, CH_3); 2.46 (3H, s, CH_3); 2.63–2.69 (1H, m, H-2); 3.87−3.93 (1H, m, H-1); 4.27 (1H, td, *J* = 4.5, *J* = 3.1, H-3); 5.31 (1H, d, *J* = 4.6, H-4 pyrazole); 5.99 (1H, br. s, OH); 6.41 (2H, d, *J* = 9.2, H Ph); 6.99 (2H, d, *J* = 6.1, H Ph); 7.31–7.36 (3H, m, H Ph); 7.43 (2H, d, *J* = 9.0, H Ph); 8.06 (1H, br. s, NH).

^{13}C NMR spectrum (75 MHz), δ, ppm: 14.1, 14.4 (C-3 pyrazole-$\underline{C}H_3$ and C-5 pyrazole-$\underline{C}H_3$); 51.0 (C-2); 58.5 (C-3); 60.7 (C-1); 111.6 (C-4 pyrazole); 114.7, 126.7, 126.9 (NH-(C-2−6 Ph)); 128.5, 128.6, 128.8 (3-(C-2−6 Ph)); 129.0 (3-(C-1 Ph)); 140.0 (C-5 pyrazole); 145.4 (NH-(C-1 Ph)); 152.8 (C-3 pyrazole); 174.0 (C=O). Found, *m*/*z*: 382.1365 $[M–H]^–$. $C_{21}H_{21}ClN_3O_2$ Calculated, *m*/*z*: 382.1401.

2-(3,5-Dimethylpyrazol-1-ylcarbonyl)-3-(3-cyanophenylamino)-3-phenylpropan-1-ol (**11**): Yield 42.23 mg (84%), colorless oily liquid. IR spectrum, ν, $cm^{–1}$: 3376, 3030, 2927, 2227, 1720, 1602, 1583, 1489, 1382, 1329, 1043, 950, 780, 702, 682. ^1H NMR spectrum (300 MHz), δ, ppm (*J*, Hz): 2.36 (3H, s, CH_3); 2.45 (3H, s, CH_3); 2.65–2.69 (1H, m, H-2); 3.85–4.10 (1H, m, H-1); 4.24 (1H, d, *J* = 3.9, H-3); 5.37 (1H, d, *J* = 4.2, H-4 pyrazole); 6.02 (1H, br. s, OH); 6.60 (1H, s, H Ph);6.87 (1H, d, *J* = 6.0, H Ph); 7.08–7.41 (7H, m, H Ph); 8.04 (1H, br. s, NH). ^{13}C NMR spectrum (75 MHz), δ, ppm: 14.0, 14.4 (C-3 pyrazole-$\underline{C}H_3$ and C-5 pyrazole-$\underline{C}H_3$); 50.8 (C-2); 58.1 (C-3); 60.6 (C-1); 111.8 (NH-(C-2, 6 Ph)); 112.6 (C-4 pyrazole); 115.5 (NH-(C-3 Ph)); 118.1 (NH-(C-3 Ph-$\underline{C}$N); 119.4, 120.8, 126.6 (NH-(C-4, 5 Ph)); 127.7, 128.8, 129.6 (3-(C-2−6 Ph)); 139.3 (3-(C-1 Ph)); 144.5 (C-5 pyrazole); 147.2 (NH-(C-1 Ph)); 153.1 (C-3 pyrazole); 173.8 (C=O). Found, *m*/*z*: 373.1701 $[M–H]^–$. $C_{22}H_{21}N_4O_2$ Calculated, *m*/*z*: 373.1743.

2-(3,5-Dimethylpyrazol-1-ylcarbonyl)-3-(phenylamino)-3-(9-anthryl)propan-1-ol (**12**): Yield 30.13 mg (60%), colorless oily liquid. IR spectrum, ν, $cm^{–1}$: 3053, 2916, 2848, 2250, 1715, 1602, 1498, 1379, 1321, 1158, 1030, 908, 733. ^1H NMR spectrum (300 MHz), δ, ppm (*J*, Hz): 2.04 (3H, s, CH_3); 2.07 (3H, s, CH_3); 2.65–2.69 (1H, m, H-2); 4.27–4.44 (2H, m, H-1); 5.18 (1H, dt, *J* = 8.8, *J* = 4.9, H-3); 5.30 (1H, d, *J* = 4.5, H-4 pyrazole); 5.67

(1H, br. s, OH); 6.58-6.68 (4H, m, H Ph); 6.98 (1H, d, *J* = 6.4, H Ph); 7.44–7.55 (5H, m, H Anthryl); 7.96 (2H, d, *J* = 9.6, H Anthryl); 8.34 (1H, br. s, NH); 8.89–8.96 (2H, m, H Anthryl). ^{13}C NMR spectrum (75 MHz), δ, ppm: 13.4, 13.8 (C-3 pyrazole-$\underline{C}H_3$ and C-5 pyrazole-$\underline{C}H_3$); 51.6 (C-2); 55.2 (C-3); 62.2 (C-1); 111.3 (C-4 pyrazole); 113.6, 119.4 (NH-(C-2, 4, 6 Ph)); 122.1, 124.6, 128.7, 129.1, 130.0 (3-(C-2−5, 8, 11−14 Anthryl)); 133.6 (NH-(C-3, 5 Ph)); 138.1, 140.9, 143.8 (3-(C Anthryl)); 144.4 (C-5 pyrazole); 148.0 (NH-(C-1 Ph)); 152.0 (C-3 pyrazole); 170.1 (C=O). Found, *m*/*z*: 488.3088 $[M+K]^+$. $C_{29}H_{27}N_3O_2K$ Calculated, *m*/*z*: 488.3063.

4-Hydroxymethyl-2,3-diphenylisoxazolidine (**13**): Yield 33.80 mg (92%), colorless oily liquid. IR spectrum, ν, cm^{-1}: 3393, 3060, 3028, 2959, 2924, 2871, 1597, 1488, 1451, 1261, 1073, 1029, 800, 754, 697. ^{1}H NMR spectrum (300 MHz), δ, ppm (*J*, Hz): 1.70–1.73 (1H, m, H-4); 2.95 (1H, d, *J* = 9.2, H-3); 3.68–3.81 (2H, m, $C\underline{H}_2OH$); 3.97 (1H, br. s, $CH_2O\underline{H}$); 4.31–4.38 (2H, m, H-5); 6.92–6.99 (3H, m, H Ph); 7.23 (2H, d, *J* = 9.4, H Ph); 7.26–7.41 (3H, m, H Ph); 7.54 (2H, d, *J* = 6.2, H Ph). ^{13}C NMR spectrum (75 MHz), δ, ppm: 56.7 (C-4); 62.7 (CH_2OH); 69.3 (C-5); 72.2 (C-3); 114.7, 121.6, 126.6 (2-(C-2−6 Ph)); 127.5, 128.8, 128.9 (3-(C-2−6 Ph)); 142.1 (3-(C-1 Ph)); 151.1 (2-(C-1 Ph)). Found, *m*/*z*: 210.0890 $[M–COOH]^-$. $C_{16}H_{17}NO_2$ Calculated, *m*/*z*: 210.0859.

2-(4-Methylphenyl)-4-hydroxymethyl-3-phenylisoxazolidine (**14**): Yield 35.02 mg (94%), colorless oily liquid. IR spectrum, ν, cm^{-1}: 3399, 3027, 2922, 2870, 1611, 1506, 1453, 1342, 1261, 1042, 813, 700. ^{1}H NMR spectrum (300 MHz), δ, ppm (*J*, Hz): 2.04–2.07 (1H, m, H-4); 2.28 (3H, s, 2-(4 Ph-$C\underline{H}_3$)); 2.87 (1H, d, *J* = 6.0, H-3); 3.69–3.94 (2H, m, $C\underline{H}_2OH$); 3.98 (1H, br. s, $CH_2O\underline{H}$); 4.28–4.35 (2H, m, H-5); 6.89 (2H, d, *J* = 9.4, H Ph);

7.04 (2H, d, *J* = 9.1, H Ph); 7.32–7.42 (3H, m, H Ph); 7.52 (2H, d, *J* = 6.2, H Ph). ^{13}C NMR spectrum (75 MHz), δ, ppm: 20.6 (2-(C-4 Ph-C̲H$_3$)); 56.7 (C-4); 62.8 (CH$_2$OH); 69.3 (C-5); 72.5 (C-3); 115.1, 126.7 (2-(C-2−6 Ph)); 127.5, 128.8, 129.3 (3-(C-2−6 Ph)); 131.3 (2-(C-4 Ph)); 142.0 (3-(C-1 Ph)); 148.7 (2-(C-1 Ph)). Found, *m*/*z*: 308.1292 [M+K]$^+$. $C_{17}H_{19}NO_2K$ Calculated, *m*/*z*: 308.1316.

2-(4-Ethylphenyl)-4-hydroxymethyl-3-phenylisoxazolidine (**15**): Yield 35.09 mg (93%), colorless oily liquid. IR spectrum, ν, cm^{-1}: 3403, 3027, 2961, 2928, 2871, 1610, 1506, 1454, 1374, 1268, 1045, 830, 761, 700. ^{1}H NMR spectrum (300 MHz), δ, ppm (*J*, Hz): 1.20 (3H, t, *J* = 7.6, 2-(4 Ph-CH$_2$CH̲$_3$)); 1.65–1.69 (1H, m, H-4); 2.57–2.59 (2H, m, 2-(4 Ph-CH̲$_2$CH$_3$)); 2.89 (1H, d, *J* = 6.4, H-3); 3.71–3.78 (2H, m, CH̲$_2$OH); 3.98 (1H, br. s, CH$_2$OH̲); 4.29–4.33 (2H, m, H-5); 6.91 (2H, d, *J* = 9.1, H Ph); 7.07 (2H, d, *J* = 9.0, H Ph); 7.31–7.43 (3H, m, H Ph); 7.54 (2H, d, *J* = 6.4, H Ph). ^{13}C NMR spectrum (75 MHz), δ, ppm: 15.7 (2-(C-4 Ph-CH$_2$C̲H$_3$)); 28.0 (2-(C-4 Ph-C̲H$_2$CH$_3$)); 56.7 (C-4); 62.9 (CH$_2$OH); 69.3 (C-5); 72.3 (C-3); 115.1, 126.7 (2-(C-2−6 Ph)); 127.5, 128.1, 128.8 (3-(C-2−6 Ph)); 137.7 (3-(C-1 Ph)); 142.1 (2-(C-4 Ph)); 148.9 (2-(C-1 Ph)). Found, *m*/*z*: 248.9625 [M–Cl]$^-$. $C_{18}H_{21}NO_2$ Calculated, *m*/*z*: 248.9572.

2-(4-Chlorophenyl)-4-hydroxymethyl-3-phenylisoxazolidine (**16**): Yield 34.89 mg (92%), colorless oily liquid. IR spectrum, ν, cm^{-1}: 3406, 3029, 2924, 2873, 1592, 1487, 1454, 1279, 1175, 1093, 1006, 824, 752, 700, 507. ^{1}H NMR spectrum (300 MHz), δ, ppm (*J*, Hz): 1.68–1.72 (1H, m, H-4); 2.90 (1H, d, *J* = 8.2, H-3); 3.72–3.77 (2H, m, CH̲$_2$OH); 3.96 (1H, br. s, CH$_2$OH̲); 4.30–4.35 (2H, m, H-5); 6.89 (2H, d, *J* = 9.3, H Ph); 7.18 (2H, d, *J* = 9.7, H Ph); 7.32–7.43 (3H, m, H Ph); 7.51 (2H, d, *J* = 7.6, H Ph). ^{13}C NMR spectrum

(75 MHz), δ, ppm: 56.6 (C-4); 62.5 (CH_2OH); 69.3 (C-5); 72.2 (C-3); 116.1, 126.6 (2-(C-2−6 Ph)); 126.7, 127.7, 128.7 (3-(C-2−6 Ph)); 128.9 (2-(C-4 Ph)); 141.6 (3-(C-1 Ph)); 149.6 (2-(C-1 Ph)). Found, *m*/*z*: 254.0707 $[M–Cl]^-$. $C_{16}H_{16}ClNO_2$ Calculated, *m*/*z*: 254.0720.

2-(3-Cyanophenyl)-4-hydroxymethyl-3-phenylisoxazolidine (**17**): Yield 35.38 mg (94%), colorless oily liquid. IR spectrum, ν, cm^{-1}: 3432, 3063, 3030, 2924, 2873, 2229, 1596, 1576, 1477, 1282, 1046, 954, 789, 701, 684. ^1H NMR spectrum (300 MHz), δ, ppm (*J*, Hz): 2.10–2.13 (1H, m, H-4); 2.89 (1H, d, *J* = 6.4, H-3); 3.74–3.93 (2H, m, $C\underline{H}_2OH$); 3.97 (1H, br. s, $CH_2O\underline{H}$); 4.32–4.39 (2H, m, H-5); 7.10 (2H, d, *J* = 6.2, H Ph); 7.16–7.52 (7H, m, H Ph). ^{13}C NMR spectrum (75 MHz), δ, ppm: 56.6 (C-4); 61.9 (CH_2OH); 69.4 (C-5); 71.5 (C-3); 112.5 (2-(C-3 Ph)); 117.4 (2-(C-2, 6 Ph)); 118.7 (2-(C-3 Ph-$\underline{C}$N)); 119.1, 124.7, 126.4 (2-(C-4, 5 Ph)); 127.9, 129.1, 129.6 (3-(C-2, 4−6 Ph)); 140.4 (3-(C-1 Ph)); 151.5 (2-(C-1 Ph)). Found, *m*/*z*: 245.0666 $[M–Cl]^-$. $C_{17}H_{16}N_2O_2$ Calculated, *m*/*z*: 245.0612.

3-(9-Anthryl)-4-hydroxymethyl-2-phenylisoxazolidine (**18**): Yield 36.14 mg (91%), colorless oily liquid. IR spectrum, ν, cm^{-1}: 3455, 2917, 2848, 2253, 1730, 1597, 1486, 1374, 1248, 1045, 908, 732. ^1H NMR spectrum (300 MHz), δ, ppm (*J*, Hz): 1.45–1.49 (1H, m, H-4); 3.60–3.82 (2H, m, $C\underline{H}_2OH$); 4.23–4.29 (2H, m, H-5); 4.74 (1H, dd, *J* = 8.3, *J* = 6.7, H-3); 5.93 (1H, br. s, $CH_2O\underline{H}$); 6.87–6.95 (3H, m, H Ph); 7.15 (2H, dd, *J* = 8.6, *J* = 7.2, H Ph); 7.49–7.56 (4H, m, H Anthryl); 8.07 (2H, d, *J* = 8.5, H Anthryl); 8.47 (2H, d, *J* = 10.8, H Anthryl); 9.25 (1H, s, H Anthryl). ^{13}C NMR spectrum (75 MHz), δ, ppm: 55.7 (C-4); 60.5 (CH_2OH); 68.1 (C-5); 71.2 (C-3); 114.5 (2-(C-2, 6 Ph)); 122.3, 124.6, 125.2, 125.8 (3-(C-2−5, 11−14 Anthryl)); 126.3, 126.7 (2-(C-3−5 Ph)); 128.8, 129.2, 129.8, 130.5 (3-(C Anthryl)); 153.2 (2-(C-1 Ph)). Found, *m*/*z*: 310.1579 $[M–COOH]^-$. $C_{24}H_{21}NO_2$ Calculated,

m/z: 310.1572.

2-(2-(4-phenyl)-3-phenylisoxazolidin-4-yl)propan-2-ol (**19**): 29.82 mg Yield (73%), colorless oily liquid. IR spectrum, ν, cm^{-1}: 3435, 3061, 3029, 2972, 2926, 2873, 1598, 1489, 1452, 1370, 1239, 1143, 1029, 945, 754, 697. ^1H NMR spectrum (300 MHz), δ, ppm (*J*, Hz): 1.18, 1.24 (6H, s, 2×C-($C\underline{H}_3)_2$); 1.52–1.56 (1H, m, H-4); 2.87 (1H, ddd, *J* = 7.6, *J* = 6.2, *J* = 4.8, H-3); 4.07–4.12 (1H, m, H-5); 4.29–4.34 (1H, m, H-5); 4.60 (1H, br. s, OH); 7.20–7.23 (3H, m, H Ph); 7.30–7.33 (3H, m, H Ph); 7.41 (2H, d, *J* = 9.5, H Ph); 7.59 (2H, d, *J* = 4.5, H Ph). ^{13}C NMR spectrum (75 MHz), δ, ppm: 27.8, 29.1 (C for quaternary-($\underline{C}H_3)_2$); 65.1 (C-5); 65.2 (C-4); 68.3 (C for quaternary); 71.4 (C-3); 114.7, 121.5, 127.2 (2-(C-2−6 Ph)); 127.3, 128.7, 128.8 (3-(C-2−6 Ph)); 143.6 (3-(C-1 Ph)); 150.5 (2-(C-1 Ph)). Found, m/z: 248.9623 $[M–Cl]^-$. $C_{18}H_{21}NO_2$ Calculated, m/z: 248.9575.

2-(2-(4-Methylphenyl)-3-phenylisoxazolidin-4-yl)propan-2-ol (**20**): Yield 30.90 mg (75%), colorless oily liquid. IR spectrum, ν, cm^{-1}: 3437, 3027, 2972, 2923, 2872, 1611, 1507, 1454, 1371, 1240, 1143, 1080, 1029, 947, 813, 700. ^1H NMR spectrum (300 MHz), δ, ppm (*J*, Hz): 1.17, 1.24 (6H, s, (6H, s, 2×C-($C\underline{H}_3)_2$); 1.54–1.57 (1H, m, H-4); 2.27 (3H, s, 2-(4 Ph-$C\underline{H}_3$)); 2.83 (1H, ddd, *J* = 7.7, *J* = 5.9, *J* = 4.9, H-3); 4.07–4.12 (1H, m, H-5); 4.28–4.33 (1H, m, H-5); 4.51 (1H, br. s, OH); 6.88 (2H, d, *J* = 9.5, H Ph); 7.03 (2H, d, *J* = 6.2, H Ph); 7.29–7.41 (3H, m, H Ph); 7.57 (2H, d, *J* = 9.2, H Ph). ^{13}C NMR spectrum (75 MHz), δ, ppm: 20.6 (2-(C-4 Ph-$\underline{C}H_3$)); 27.9, 29.0 (C for quaternary-($\underline{C}H_3)_2$); 65.0 (C-5); 67.9 (C-4); 68.3 (C for quaternary); 71.8 (C-3); 115.2, 127.3 (2-(C-2,3, 5, 6 Ph)); 127.4, 128.7, 129.3 (3-(C-2−6 Ph)); 131.1 (2-(C-4 Ph)); 143.5 (3-(C-1 Ph)); 148.1 (2-(C-1 Ph)). Found, m/z: 252.1398 $[M–COOH]^-$. $C_{19}H_{23}NO_2$ Calculated, m/z: 252.1416.

2-(2-(4-Ethylphenyl)-3-phenylisoxazolidin-4-yl)propan-2-ol (**21**): Yield 31.15 mg (75%), colorless oily liquid. IR spectrum, ν, cm^{-1}: 3442, 3028, 2965, 2929, 2872, 1611, 1507, 1454, 1371, 1236, 1140, 935, 829, 700. ^{1}H NMR spectrum (300 MHz), δ, ppm (*J*, Hz): 1.17–1.22 (9H, m, 2-(4 Ph-$CH_2C\underline{H}_3$)) and C-($C\underline{H}_3$)$_2$); 1.55–1.58 (1H, m, H-4); 2.59 (1H, t, *J* = 8.2, H-3); 2.61 (2H, q, *J* = 7.6, 2-(4 Ph-$C\underline{H}_2CH_3$)); 4.08–4.13 (1H, m, H-5); 4.28–4.34 (1H, m, H-5); 4.53 (1H, br. s, OH); 6.90 (2H, d, *J* = 6.0, H Ph); 7.06 (2H, d, *J* = 9.2, H Ph); 7.30–7.41 (3H, m, H Ph); 7.58 (2H, d, *J* = 9.0, H Ph). ^{13}C NMR spectrum (75 MHz), δ, ppm: 15.6 (2-(C-4 Ph-$CH_2\underline{C}H_3$)); 27.9, 28.0 (C for quaternary-($\underline{C}H_3$)$_2$); 29.0 (2-(C-4 Ph-$\underline{C}H_2CH_3$)); 65.0 (C-5); 68.2 (C-4); 71.2 (C for quaternary); 71.7 (C-3); 115.2, 127.3 (2-(C-2,3, 5, 6 Ph)); 127.4, 128.0, 128.7 (3-(C-2−6 Ph)); 137.6 (3-(C-1 Ph)); 143.6 (2-(C-4 Ph)); 148.3 (2-(C-1 Ph)). Found, *m*/*z*: 350.1580 $[M+K]^+$. $C_{20}H_{25}NO_2K$ Calculated, *m*/*z*: 350.1585.

2-(2-(4-Chlorophenyl)-3-phenylisoxazolidin-4-yl)propan-2-ol (**22**): Yield 30.01 mg (72%), colorless oily liquid. IR spectrum, ν, cm^{-1}: 3451, 2971, 2925, 1592, 1487, 1370, 1235, 1093, 946, 823, 700. ^{1}H NMR spectrum (300 MHz), δ, ppm (*J*, Hz): 1.18, 1.24 (6H, s, (6H, s, 2×C-($C\underline{H}_3$)$_2$); 1.61–1.63 (1H, m, H-4); 2.86 (1H, ddd, *J* = 7.6, *J* = 6.1, *J* = 4.8, H-3); 4.05–4.10 (1H, m, H-5); 4.28–4.30 (1H, m, H-5); 4.54 (1H, br. s, OH); 6.89 (2H, d, *J* = 9.5, H Ph); 7.17 (2H, d, *J* = 9.2, H Ph); 7.30–7.41 (3H, m, H Ph); 7.54 (2H, d, *J* = 6.1, H Ph). ^{13}C NMR spectrum (75 MHz), δ, ppm: 27.9, 29.1 (C for quaternary-($\underline{C}H_3$)$_2$); 62.9 (C-5); 64.9 (C-4); 68.4 (C for quaternary); 71.5 (C-3); 116.1, 126.5 (2-(C-2, 3, 5, 6 Ph)); 127.2, 127.5, 128.6 (3-(C-2−6 Ph)); 128.9 (2-(C-4 Ph)); 143.1 (3-(C-1 Ph)); 149.0 (2-(C-1 Ph)). Found, *m*/*z*: 356.1271 $[M+K]^+$. $C_{18}H_{20}ClNO_2K$ Calculated, *m*/*z*: 356.1183.

2-(2-(3-Cyanophenyl)-3-phenylisoxazolidin-4-yl)propan-2-ol (**23**): Yield 30.27 mg (73%), colorless oily liquid. IR spectrum, ν, cm^{-1}: 3452, 2923, 2226, 1596, 1477, 1371, 1151, 1026, 935, 886, 784, 690. ^{1}H NMR spectrum (300 MHz), δ, ppm (*J*, Hz): 1.19, 1.26 (6H, s, 2×C-(C<u>H</u>$_3$)$_2$); 1.55–1.57 (1H, m, H-4); 2.87 (1H, ddd, *J* = 7.5, *J* = 6.4, *J* = 4.6, H-3); 4.04–4.09 (1H, m, H-4); 4.29–4.35 (1H, m, H-4); 4.62 (1H, br. s, OH); 7.09 (1H, *J* = 6.3, H Ph); 7.16 (1H, *J* = 6.0, H Ph); 7.30–7.42 (5H, m, H Ph); 7.54 (2H, *J* = 9.1, H Ph). ^{13}C NMR spectrum (75 MHz), δ, ppm: 27.9, 29.3 (C for quaternary-(<u>C</u>H$_3$)$_2$); 65.0 (C-5); 68.4 (C-4); 70.7 (C for quaternary); 70.8 (2-(C-3 Ph)); 112.5 (2-(C-3 Ph-<u>C</u>N)); 117.3, 118.6, 119.1, 124.5 (2-(C-2, 3, 5, 6 Ph)); 127.0, 127.6, 129.0, 129.5 (3-(C-2−6 Ph)); 142.8 (3-(C-1 Ph)); 150.9 (2-(C-1 Ph)). Found, *m*/*z*: 347.1643 $[M+K]^{+}$. $C_{19}H_{20}N_2O_2K$ Calculated, *m*/*z*: 347.1625.

2-(2-(3-Phenyl)-3-(9-anthryl)isoxazolidin-4-yl)propan-2-ol (**24**): Yield 30.02 mg (70%), colorless oily liquid. IR spectrum, ν, cm^{-1}: 3447, 2984, 2926, 2853, 2254, 1732, 1655, 1465, 1374, 1248, 1046, 908, 734, 649. ^{1}H NMR spectrum (300 MHz), δ, ppm (*J*, Hz): 1.15, 1.26 (3H, s, 2×C-(C<u>H</u>$_3$)$_2$); 3.63–3.65 (1H, m, H-4); 4.36–4.39 (2H, dd, *J* = 9.5, *J* = 8.4, H-5); 4.71 (1H, dd, *J* = 8.4, *J* = 7.2, H-3); 6.14 (1H, d, *J* = 8.7, OH); 6.90–6.99 (3H, m, H Ph); 7.10–7.13 (2H, m, H Ph); 7.49–7.62 (4H, m, H Anthryl); 8.05–8.08 (2H, m, H Anthryl); 8.50–8.55 (2H, m, H Anthryl); 9.24 (1H, d, *J* = 9.0, H Anthryl). ^{13}C NMR spectrum (75 MHz), δ, ppm: 27.8, 29.7 (C for quaternary-(<u>C</u>H$_3$)$_2$); 62.7 (C-5); 67.6 (C-4); 70.0 (C for quaternary); 70.2 (C-3); 115.1 (2-(C-2, 6 Ph)); 124.4, 125.0, 125.8, 126.5 (3-(C-2−5, 11−14 Anthryl)); 126.6, 128.7 (2-(C-3−5 Ph)); 129.8, 131.5, 132.2, 132.2 (3-(C Anthryl)); 152.9 (2-(C-1 Ph)). Found, *m*/*z*: 348.1645 $[M–Cl]^{-}$. $C_{26}H_{25}NO_2$ Calculated, *m*/*z*:

348.1685.

4-Acetyl-2,3-diphenylisoxazolidine (**25**): Yield 26.55 mg (69%), colorless oily liquid. IR spectrum, ν, cm^{-1}: 2954, 2916, 2848, 1736, 1598, 1459, 1373, 1244, 1176, 1045, 909, 733, 698. ^{1}H NMR spectrum (300 MHz), δ, ppm (*J*, Hz): 2.17 (3H, s, $COCH_3$); 3.71 (1H, q, *J* = 6.1, H-4); 4.20–4.25 (1H, m, H-5), 4.46 (1H, t, *J* = 6.7, H-5), 4.94 (1H, d, *J* = 6.1, H-3); 6.94–7.00 (3H, m, H Ph); 7.22 (2H, d, *J* = 3.2, H Ph); 7.33–7.44 (3H, m, H Ph); 7.53 (2H, d, *J* = 6.2, H Ph). ^{13}C NMR spectrum (75 MHz), δ, ppm: 29.7 (CO<u>C</u>H_3); 67.0 (C-4); 68.3 (C-5); 71.2 (C-3); 115.2, 122.2, 126.7 (2-(C-2−6 Ph)); 127.8, 128.8, 129.0 (3-(C-2−6 Ph)); 141.4 (3-(C-1 Ph)); 150.5 (2-(C-1 Ph)); 203.9 (C=O). Found, *m*/*z*: 268.1337 $[M+H]^+$. $C_{17}H_{18}NO_2$ Calculated, *m*/*z*: 268.1259.

4-Acetyl-2-(4-methylphenyl)-3-phenylisoxazolidine (**26**): Yield 27.24 mg (70%), colorless oily liquid. IR spectrum, ν, cm^{-1}: 2917, 2848, 1734, 1507, 1461, 1374, 1247, 1046, 908, 733, 649. ^{1}H NMR spectrum (300 MHz), δ, ppm (*J*, Hz): 2.16 (3H, s, $COCH_3$); 2.28 (3H, s, 2-(4 Ph-C<u>H</u>$_3$)); 3.70 (1H, q, *J* = 6.2, H-4); 4.22–4.25 (1H, m, H-5), 4.44 (1H, t, *J* = 9.3 Hz, H-5), 4.84 (1H, d, *J* = 6.0, H-3); 6.90 (2H, d, *J* = 9.3, H Ph); 7.04 (2H, d, *J* = 9.1, H Ph); 7.32–7.43 (3H, m, H Ph); 7.52 (2H, d, *J* = 9.0, H Ph). ^{13}C NMR spectrum (75 MHz), δ, ppm: 20.6 (2-(C-4 Ph-<u>C</u>H_3)); 29.7 (CO<u>C</u>H_3); 66.8 (C-4); 68.2 (C-5); 71.6 (C-3); 115.8, 126.8 (2-(C-2, 3, 5, 6 Ph)); 127.8, 128.9, 129.3 (3-(C-2−6 Ph)); 132.0 (2-(C-4 Ph)); 141.2 (3-(C-1 Ph)); 147.9 (2-(C-1 Ph)); 204.1 (C=O). Found, *m*/*z*: 282.1496 $[M+H]^+$. $C_{18}H_{20}NO_2$ Calculated, *m*/*z*: 282.1416.

4-Acetyl-2-(4-ethylphenyl)-3-phenylisoxazolidine (**27**): Yield 27.53 mg (70%), colorless oily liquid. IR spectrum, ν, cm^{-1}: 2957, 2916, 2848, 1735, 1508, 1462, 1373, 1245,

1046, 909, 733, 648. ^{1}H NMR spectrum (300 MHz), δ, ppm (*J*, Hz): 1.21 (3H, t, *J* = 6.8 Hz, $COCH_3$), 2.16 (3H, s, 2-(4 Ph-$CH_2C\underline{H}_3$)); 2.54–2.62 (2H, m, 2-(4 Ph-$C\underline{H}_2CH_3$)); 3.70 (1H, q, *J* = 6.1, H-4); 4.21–4.27 (1H, m, H-5), 4.44 (1H, t, *J* = 6.5 Hz, H-5), 4.86 (1H, d, *J* = 6.3, H-3); 6.92 (2H, d, *J* = 6.4, H Ph); 7.07 (2H, d, *J* = 9.0, H Ph); 7.30–7.43 (3H, m, H Ph); 7.53 (2H, d, *J* = 9.4, H Ph). ^{13}C NMR spectrum (75 MHz), δ, ppm: 15.6 (2-(C-4 Ph-$CH_2\underline{C}H_3$)); 28.0 (2-(C-4 Ph-$\underline{C}H_2CH_3$)); 29.7 ($CO\underline{C}H_3$); 66.9 (C-4); 68.2 (C-5); 71.4 (C-3); 115.7, 126.8 (2-(C-2, 3, 5, 6 Ph)); 127.8, 128.1, 128.9 (3-(C-2−6 Ph)); 138.4 (3-(C-1 Ph)); 141.3 (2-(C-4 Ph)); 148.1 (2-(C-1 Ph)); 204.1 (C=O). Found, *m*/*z*: 296.1649 $[M+H]^+$. $C_{19}H_{22}NO_2$ Calculated, *m*/*z*: 296.1572.

4-Acetyl-2-(4-chlorophenyl)-3-phenylisoxazolidine (**28**): Yield 26.86 mg (68%), colorless oily liquid. IR spectrum, ν, cm^{-1}: 2925, 2360, 2254, 1734, 1488, 1437, 1374, 1247, 1175, 1046, 908, 825, 733, 649. ^{1}H NMR spectrum (300 MHz), δ, ppm (*J*, Hz): 2.18 (3H, s, $COCH_3$); 3.74 (1H, q, *J* = 3.2, H-4); 4.18–4.23 (1H, m, H-5), 4.45 (1H, t, *J* = 9.4 Hz, H-5), 4.86 (1H, d, *J* = 6.3, H-3); 6.90 (2H, d, *J* = 9.2, H Ph); 7.19 (2H, d, *J* = 9.0, H Ph); 7.34–7.43 (3H, m, H Ph); 7.49 (2H, d, *J* = 6.3, H Ph). ^{13}C NMR spectrum (75 MHz), δ, ppm: 29.8 ($CO\underline{C}H_3$); 66.7 (C-4); 68.4 (C-5); 71.2 (C-3); 116.6, 126.7 (2-(C-2, 3, 5, 6 Ph)); 127.3, 128.0, 128.7 (3-(C-2−6 Ph)); 129.1 (2-(C-4 Ph)); 140.9 (3-(C-1 Ph)); 148.9 (2-(C-1 Ph)); 203.8 (C=O). Found, *m*/*z*: 302.0953 $[M+H]^+$. $C_{17}H_{17}ClNO_2$ Calculated, *m*/*z*: 302.0870.

2,3-Diphenylisoxazolidin-4-ylcarbohydrazide (**29**): Yield 37.52 mg (92%), yellowish solid. mp 195.4-196.0 °C; IR spectrum, ν, cm^{-1}: 3279, 3029, 2925, 2871, 1735, 1663, 1597, 1487, 1452, 1352, 1251, 1089, 1027, 914, 755, 697. ^{1}H NMR spectrum (300 MHz), δ, ppm (*J*, Hz): 3.28–3.31 (1H, m, H-4); 3.76–3.90 (2H, m, H-5); 4.27 (1H, d, *J* = 7.2, H-3); 4.36

(2H, br. s, NH_2); 6.95–6.99 (3H, m, H Ph); 7.19 (2H, dd, *J* = 8.4, *J* = 7.3, H Ph); 7.21–7.23 (3H, m, H Ph); 7.37–7.50 (2H, m, H Ph); 9.11 (1H, br. s, NH). ^{13}C NMR spectrum (75 MHz), δ, ppm: 59.1 (C-4); 69.6 (C-5); 73.4 (C-3); 115.1, 122.3, 126.5 (2-(C-2−6 Ph)); 128.1, 128.8, 129.1 (3-(C-2−6 Ph)); 140.7 (3-(C-1 Ph)); 150.6 (2-(C-1 Ph)); 170.6 (C=O). Found, *m*/*z*: 282.1268 $[M–H]^-$. $C_{16}H_{16}N_3O_2$ Calculated, *m*/*z*: 282.1321.

2-(4-Methylphenyl)-3-phenylisoxazolidin-4-ylcarbohydrazide (**30**): Yield 38.26 mg (93%), yellowish solid. mp 197.1-197.7 °C; IR spectrum, ν, cm^{-1}: 3278, 3029, 2922, 2855, 1655, 1612, 1506, 1453, 1384, 1253, 1024, 908, 812, 732, 700. ^1H NMR spectrum (300 MHz), δ, ppm (*J*, Hz): 2.27 (3H, s, 2-(4 Ph-$\underline{C}H_3$)); 3.28–3.30 (1H, m, H-4); 3.74–3.89 (2H, m, H-5); 4.32 (1H, d, *J* = 7.4, H-3); 4.75 (2H, br. s, NH_2); 6.89 (2H, d, *J* = 9.5, H Ph); 7.04 (2H, d, *J* = 4.5, H Ph); 7.36–7.49 (5H, m, H Ph); 9.00 (1H, br. s, NH). ^{13}C NMR spectrum (75 MHz), δ, ppm: 20.6 (2-(C-4 Ph-$\underline{C}H_3$)); 59.0 (C-4); 69.4 (C-5); 73.8 (C-3); 115.8, 126.7 (2-(C-2, 3, 5, 6 Ph)); 128.1, 129.1, 129.3 (3-(C-2−6 Ph)); 132.1 (2-(C-4 Ph)); 140.5 (3-(C-1 Ph)); 148.0 (2-(C-1 Ph)); 170.6 (C=O). Found, *m*/*z*: 296.1421 $[M–H]^-$. $C_{17}H_{18}N_3O_2$ Calculated, *m*/*z*: 296.1477.

2-(4-Ethylphenyl)-3-phenylisoxazolidin-4-ylcarbohydrazide (**31**): Yield 39.39 mg (95%), yellowish solid. mp 198.5-199.1 °C; IR spectrum, ν, cm^{-1}: 3323, 3275, 3028, 2924, 2870, 1642, 1522, 1503, 1454, 1254, 1120, 1022, 905, 830, 733, 699, 549. ^1H NMR spectrum (300 MHz), δ, ppm (*J*, Hz): 1.19 (3H, t, *J* = 7.6, 2-(4 Ph-$CH_2C\underline{H}_3$)); 2.57 (2H, q, *J* = 7.6, 2-(4 Ph-$C\underline{H}_2CH_3$)); 3.29 (1H, q, *J* = 7.2, H-4); 3.97 (1H, s, H-3); 4.27–4.35 (2H, dt, *J* = 15.6, *J* = 8.0, H-5); 4.76 (2H, br. s, NH_2); 6.91 (2H, d, *J* = 9.5, H Ph); 7.07 (2H, d, *J* = 8.4, H Ph); 7.36–7.39 (3H, m, H Ph); 7.47 (2H, d, *J* = 4.5, H Ph); 9.27 (1H, br. s, NH).

^{13}C NMR spectrum (75 MHz), δ, ppm: 15.6 (2-(C-4 Ph-$CH_2\underline{C}H_3$)); 28.0 (2-(C-4 Ph-$\underline{C}H_2CH_3$)); 59.1 (C-4); 69.5 (C-5); 73.6 (C-3); 115.7, 126.7 (2-(C-2, 3, 5, 6 Ph)); 128.1, 128.7, 129.1 (3-(C-2−6 Ph)); 138.5 (3-(C-1 Ph)); 140.6 (2-(C-4 Ph)); 148.2 (2-(C-1 Ph)); 170.8 (C=O). Found, *m*/*z*: 310.1597 $[M–H]^-$. $C_{18}H_{20}N_3O_2$ Calculated, *m*/*z*: 310.1634.

2-(4-Chlorophenyl)-3-phenylisoxazolidin-4-ylcarbohydrazide (**32**): Yield 38.27 mg (92%), yellowish solid. mp 197.9-198.5 °C; IR spectrum, ν, cm^{-1}: 3317, 2923, 2854, 1734, 1663, 1487, 1456, 1252, 1094, 938, 824, 751, 701, 509. ^1H NMR spectrum (300 MHz), δ, ppm (*J*, Hz): 3.29 (1H, q, *J* = 7.5, H-4); 3.88–3.91 (2H, dt, *J* = 8.4, *J* = 8.1, H-5); 4.35 (1H, s, H-3); 4.77 (2H, br. s, NH_2); 6.89 (2H, d, *J* = 8.2, H Ph); 7.18 (2H, d, *J* = 8.4, H Ph); 7.37–7.40 (5H, m, H Ph); 9.07 (1H, br. s, NH). ^{13}C NMR spectrum (75 MHz), δ, ppm: 56.7 (C-4); 69.7 (C-5); 73.6 (C-3); 116.6, 126.5 (2-(C-2, 3, 5, 6 Ph)); 127.4, 128.3, 128.8 (3-(C-2−6 Ph)); 129.2 (2-(C-4 Ph)); 140.2 (3-(C-1 Ph)); 149.2 (2-(C-1 Ph)); 174.0 (C=O). Found, *m*/*z*: 318.1014 $[M+H]^+$. $C_{16}H_{17}ClN_3O_2$ Calculated, *m*/*z*: 318.0931.

2-(3-Cyanophenyl)-3-phenylisoxazolidin-4-ylcarbohydrazide (**33**): Yield 37.67 mg (91%), yellowish solid. mp 201.6−202.1 °C. IR spectrum, ν, cm^{-1}: 3316, 3031, 2923, 2853, 2229, 1734, 1669, 1597, 1577, 1477, 1257, 1028, 911, 790, 734, 701, 684. ^1H NMR spectrum (300 MHz), δ, ppm (*J*, Hz): 3.30–3.33 (1H, m, H-4); 3.98 (1H, d, *J* = 7.2, H-3); 4.17–4.22 (1H, m, H-5); 4.37–4.40 (1H, m, H-5); 4.88 (2H, br. s, NH_2); 7.11 (1H, ddd, *J* = 8.1, *J* = 2.4, *J* = 1.2, H Ph); 7.23–7.25 (3H, m, H Ph); 7.31–7.46 (5H, m, H Ph); 9.09 (1H, br. s, NH). ^{13}C NMR spectrum (75 MHz), δ, ppm: 59.0 (C-4); 70.2 (C-5); 73.0 (C-3); 112.7 (2-(C-3 Ph)); 117.6 (2-(C-3 Ph-$\underline{C}$N)); 118.9, 119.0, 125.3, 126.2 (2-(C-2, 4−6 Ph)); 128.4, 129.3, 129.7 (3-(C-2−6 Ph)); 142.0 (3-(C-1 Ph)); 151.3 (2-(C-1 Ph)); 169.9 (C=O). Found,

m/*z*: 307.1208 $[M–H]^-$. $C_{17}H_{15}N_4O_2$ Calculated, *m*/*z*: 307.1273.

2-Phenyl-3-(9-anthryl)isoxazolidin-4-ylcarbohydrazide (**34**): Yield 38.56 mg (90%), yellowish solid. mp 207.7–208.3 °C; IR spectrum, ν, cm^{-1}: 3276, 3053, 2925, 1670, 1623, 1596, 1487, 1451, 1386, 1250, 1087, 908, 732, 695. ^1H NMR spectrum (300 MHz), δ, ppm (*J*, Hz): 3.56–3.59 (2H, m, H-5); 3.88 (1H, d, *J* = 8.3, H-3); 4.69 (2H, br. s, NH_2); 6.33 (3H, dd, *J* = 6.3, *J* = 3.3, H Ph); 6.89 (2H, t, *J* = 7.8, H Ph); 7.52 (4H, dt, *J* = 11.3, *J* = 7.5, H Anthryl); 8.04 (2H, d, *J* = 8.2, H Anthryl); 8.34 (2H, d, *J* = 8.8, H Anthryl); 8.48 (1H, s, H Anthryl); 9.14 (1H, br. s, NH). ^{13}C NMR spectrum (75 MHz), δ, ppm: 57.3 (C-4); 70.2 (C-5); 71.2 (C-3); 114.7 (2-(C-2, 6 Ph)); 127.1, 128.9, 129.0, 129.3 (3-(C-2−4, 12−14 Anthryl)); 129.4, 129.5 (2-(C-3−5 Ph)); 129.6, 130.1, 131.3 (3-(C Anthryl)); 132.1 (3-(C-1 Ph)); 152.5 (2-(C-1 Ph)); 171.3 (C=O). Found, *m*/*z*: 340.1803 $[M–COOH]^-$. $C_{24}H_{23}N_3O_2$ Calculated, *m*/*z*: 340.1790.

Ethyl 2,3-diphenylisoxazolidin-4-ylcarboxylate (**35**): Yield 39.80 mg (93%), colorless oily liquid. IR spectrum, ν, cm^{-1}: 3061, 3030, 2962, 2926, 1734, 1598, 1489, 1451, 1374, 1260, 1181, 1093, 1024, 938, 865, 799, 754, 696. ^1H NMR spectrum (300 MHz), δ, ppm (*J*, Hz): 1.24 (3H, t, *J* = 7.1, $COOCH_2$-C<u>H</u>$_3$); 3.61 (1H, ddd, *J* = 7.9, *J* = 6.9, *J* = 5.7, H-4); 4.12–4.14 (1H, m, H-5); 4.17–4.19 (1H, m, H-5); 4.34–4.42 (2H, m, COOC<u>H</u>$_2$-CH_3); 5.04 (1H, d, *J* = 6.2, H-3); 6.97–7.03 (3H, m, H Ph); 7.24 (2H, d, *J* = 6.0, H Ph); 7.33–7.44 (3H, m, H Ph); 7.58 (2H, d, *J* = 9.2, H Ph). ^{13}C NMR spectrum (75 MHz), δ, ppm: 14.1 ($COOCH_2$-<u>C</u>H_3); 58.6 (C-4); 61.4 (COO<u>C</u>H_2-CH_3); 68.9 (C-5); 72.2 (C-3); 115.0, 122.1, 126.6 (2-(C-2−6 Ph)); 127.7, 128.8, 128.9 (3-(C-2−6 Ph)); 141.3 (3-(C-1 Ph)); 150.6 (2-(C-1 Ph)); 170.9 (C=O). Found, *m*/*z*: 296.1277 $[M–H]^-$. $C_{18}H_{18}NO_3$ Calculated, *m*/*z*: 296.1365.

Ethyl 2-(4-methylphenyl)-3-phenylisoxazolidin-4-ylcarboxylate (**36**): Yield 40.06 mg (93%), colorless oily liquid. IR spectrum, ν, cm^{-1}: 3028, 2921, 2852, 2364, 2345, 1734, 1611, 1507, 1452, 1374, 1181, 1094, 1027, 812, 700, 519. ^1H NMR spectrum (300 MHz), δ, ppm (*J*, Hz): 1.26 (3H, t, *J* = 7.4, $COOCH_2$-$C\underline{H}_3$); 2.28 (3H, s, 2-(4 Ph-$C\underline{H}_3$)); 3.56 (1H, ddd, *J* = 7.8, *J* = 6.6, *J* = 5.8, H-4); 4.13–4.16 (2H, m, H-5); 4.33–4.40 (2H, m, $COOC\underline{H}_2$-CH_3); 4.96 (1H, d, *J* = 5.8, H-3); 6.92 (2H, d, *J* = 6.5, H Ph); 7.05 (2H, d, *J* = 9.5, H Ph); 7.32–7.42 (3H, m, H Ph); 7.56 (2H, d, *J* = 9.2, H Ph). ^{13}C NMR spectrum (75 MHz), δ, ppm: 14.1 ($COOCH_2$-$\underline{C}H_3$); 20.6 (2-(C-4 Ph-$\underline{C}H_3$)); 58.5 (C-4); 61.4 ($COO\underline{C}H_2$-CH_3); 68.8 (C-5); 72.4 (C-3); 115.5, 126.7 (2-(C-2, 3, 5, 6 Ph)); 127.7, 128.8, 129.3 (3-(C-2−6 Ph)); 131.8 (2-(C-4 Ph)); 141.2 (3-(C-1 Ph)); 148.0 (2-(C-1 Ph)); 171.1 (C=O). Found, *m*/*z*: 310.1451 $[M–H]^-$. $C_{19}H_{20}NO_3$ Calculated, *m*/*z*: 310.1521.

Ethyl 2-(4-ethylphenyl)-3-phenylisoxazolidin-4-ylcarboxylate (**37**): Yield 40.31 mg (93%), colorless oily liquid. IR spectrum, ν, cm^{-1}: 3029, 2962, 2929, 2871, 1735, 1611, 1507, 1454, 1373, 1228, 1182, 1095, 1027, 938, 830, 760, 700. ^1H NMR spectrum (300 MHz), δ, ppm (*J*, Hz): 1.19–1.26 (6H, m, $COOCH_2$-$C\underline{H}_3$ and 2-(4 Ph-$CH_2C\underline{H}_3$)); 2.58–2.61 (2H, m, 2-(4 Ph-$C\underline{H}_2CH_3$)); 3.58 (1H, ddd, *J* = 7.9, *J* = 6.6, *J* = 5.8, H-4); 4.15–4.18 (2H, m, H-5); 4.36–4.41 (2H, m, $COOC\underline{H}_2$-CH_3); 5.00 (1H, d, *J* = 6.1, H-3); 6.97 (2H, d, *J* = 9.1, H Ph); 7.10 (2H, d, *J* = 9.2, H Ph); 7.34–7.44 (3H, m, H Ph); 7.59 (2H, d, *J* = 9.1, H Ph). ^{13}C NMR spectrum (75 MHz), δ, ppm: 14.1 ($COOCH_2$-$\underline{C}H_3$); 14.2 (2-(C-4 Ph-$CH_2\underline{C}H_3$)); 28.1 (2-(C-4 Ph-$\underline{C}H_2CH_3$)); 58.6 (C-4); 61.4 ($COO\underline{C}H_2$-CH_3); 68.8 (C-5); 72.3 (C-3); 115.5, 126.7 (2-(C-2, 3, 5, 6 Ph)); 127.7, 128.2, 128.3 (3-(C-2−6 Ph)); 138.5 (3-(C-1 Ph)); 141.3 (2-(C-4 Ph)); 148.3 (2-(C-1 Ph)); 171.1 (C=O). Found, *m*/*z*: 324.1605 [M–

H]$^-$. $C_{20}H_{22}NO_3$ Calculated, *m*/*z*: 324.1678.

Ethyl 2-(4-chlorophenyl)-3-phenylisoxazolidin-4-ylcarboxylate (**38**): Yield 39.96 mg (92%), colorless oily liquid. IR spectrum, ν, cm^{-1}: 2981, 2923, 2850, 1734, 1592, 1487, 1454, 1373, 1232, 1183, 1094, 1027, 938, 824, 754, 700, 508. ^{1}H NMR spectrum (300 MHz), δ, ppm (*J*, Hz): 1.25 (3H, t, *J* = 7.1, $COOCH_2$-$C\underline{H}_3$); 3.59 (1H, ddd, *J* = 7.9, *J* = 6.6, *J* = 5.6, H-4); 4.13–4.18 (2H, m, H-5); 4.20–4.41 (2H, m, $COOC\underline{H}_2$-CH_3); 4.96 (1H, d, *J* = 5.6, H-3); 6.93 (2H, d, *J* = 9.2, H Ph); 7.20 (2H, d, *J* = 9.2, H Ph); 7.38–7.43 (3H, m, H Ph); 7.53 (2H, d, *J* = 6.0, H Ph). ^{13}C NMR spectrum (75 MHz), δ, ppm: 14.1 ($COOCH_2$-$\underline{C}H_3$); 58.5 (C-4); 61.6 ($COO\underline{C}H_2$-CH_3); 68.9 (C-5); 72.2 (C-3); 116.4, 126.5 (2-(C-2, 3, 5, 6 Ph)); 127.2, 127.9, 128.7 (3-(C-2−6 Ph)); 128.9 (2-(C-4 Ph)); 140.8 (3-(C-1 Ph)); 149.1 (2-(C-1 Ph)); 170.9 (C=O). Found, *m*/*z*: 330.0917 [M–H]$^-$. $C_{18}H_{17}ClNO_3$ Calculated, *m*/*z*: 330.0975.

Ethyl 2-(3-cyanophenyl)-3-phenylisoxazolidin-4-ylcarboxylate (**39**): Yield 40.68 mg (94%), colorless oily liquid. IR spectrum, ν, cm^{-1}: 3060, 2983, 2962, 2917, 2229, 1733, 1597, 1577, 1477, 1374, 1260, 1185, 1095, 1027, 954, 791, 701, 685. ^{1}H NMR spectrum (300 MHz), δ, ppm (*J*, Hz): 1.26 (3H, t, *J* = 7.2, $COOCH_2$-$C\underline{H}_3$); 3.58 (1H, ddd, *J* = 7.7, *J* = 6.8, *J* = 5.4, H-4); 4.16–4.19 (2H, m, $COOC\underline{H}_2$-CH_3); 4.28–4.31 (1H, m, H-5); 4.40–4.43 (1H, m, H-5); 5.02 (1H, d, *J* = 6.4, H-3); 7.14 (2H, d, *J* = 8.6, H Ph); 7.23–7.45 (5H, m, H Ph); 7.53 (2H, d, *J* = 9.1, H Ph). ^{13}C NMR spectrum (75 MHz), δ, ppm: 14.1 ($COOCH_2$-$\underline{C}H_3$); 58.5 (C-4); 61.7 ($COO\underline{C}H_2$-CH_3); 69.2 (C-5); 71.8 (C-3); 112.7 (2-(C-3 Ph)); 117.7 (2-(C-3 Ph-$\underline{C}$N)); 125.3, 126.3, 128.1 (2-(C-2, 4−6 Ph)); 129.1, 129.6, 131.1 (3-(C-2−6 Ph)); 140.4 (3-(C-1 Ph)); 151.0 (2-(C-1 Ph)); 170.6 (C=O). Found, *m*/*z*: 321.1277 [M–H]$^-$. $C_{19}H_{17}N_2O_3$ Calculated, *m*/*z*: 321.1317.

Ethyl 3-(9-anthryl)-2-phenylisoxazolidin-4-ylcarboxylate (**40**): Yield 41.30 mg (93%), colorless oily liquid. IR spectrum, ν, cm^{-1}: 3448, 2916, 2848, 1730, 1597, 1488, 1374, 1301, 1244, 1189, 1021, 908, 731, 695. ^{1}H NMR spectrum (300 MHz), δ, ppm (*J*, Hz): 0.98 (3H, t, *J* = 7.1, $COOCH_2\text{-}C\underline{H}_3$); 3.98–4.02 (2H, m, $COOC\underline{H}_2\text{-}CH_3$); 4.28–4.31 (1H, m, H-5); 4.48–4.54 (1H, m, H-5); 4.87 (1H, dd, *J* = 8.4, *J* = 7.3, H-4); 6.40 (1H, d, *J* = 9.1, H Anthryl-C$\underline{H}$); 6.92–6.98 (3H, m, H Ph); 7.14–7.20 (2H, m, H Ph); 7.52–7.55 (4H, m, H Anthryl); 8.08 (2H, ddd, *J* = 8.2, *J* = 1.5, *J* = 0.7, H Anthryl); 8.52–8.54 (2H, m, H Anthryl); 9.19 (1H, s, H Anthryl). ^{13}C NMR spectrum (75 MHz), δ, ppm: 13.8 ($COOCH_2\text{-}\underline{C}H_3$); 57.4 (C-4); 61.4 ($COO\underline{C}H_2\text{-}CH_3$); 69.6 (C-5); 70.9 (C-3); 115.0 (2-(C-2, 6 Ph)); 122.1, 128.9, 129.1, 129.3, 129.3 (3-(C-2−6, 8, 10−14 Anthryl)); 129.3, 129.5 (2-(C-3−5 Ph)); 131.5, 132.2, 133.6 (3-(C Anthryl)); 152.3 (2-(C-1 Ph)); 170.6 (C=O). Found, *m*/*z*: 422.1840 $[M+Na]^+$. $C_{26}H_{25}NO_3Na$ Calculated, *m*/*z*: 422.1834.

2. ^{1}H and ^{13}C NMR spectra of compounds **1-40**.

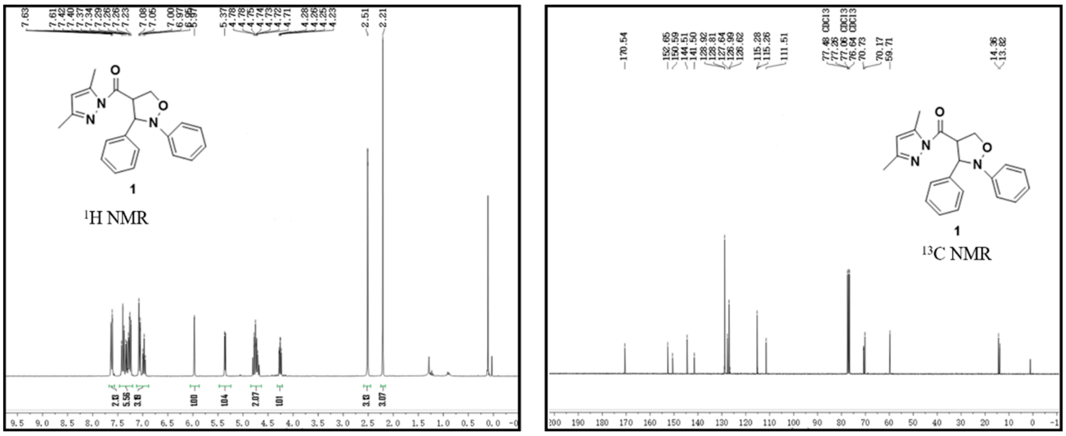

Figure S1. ^{1}H and ^{13}C NMR spectra of 4-(3,5-dimethylpyrazol-1-ylcarbonyl)-2,3-diphenylisoxazolidine (**1**) in $CDCl_3$

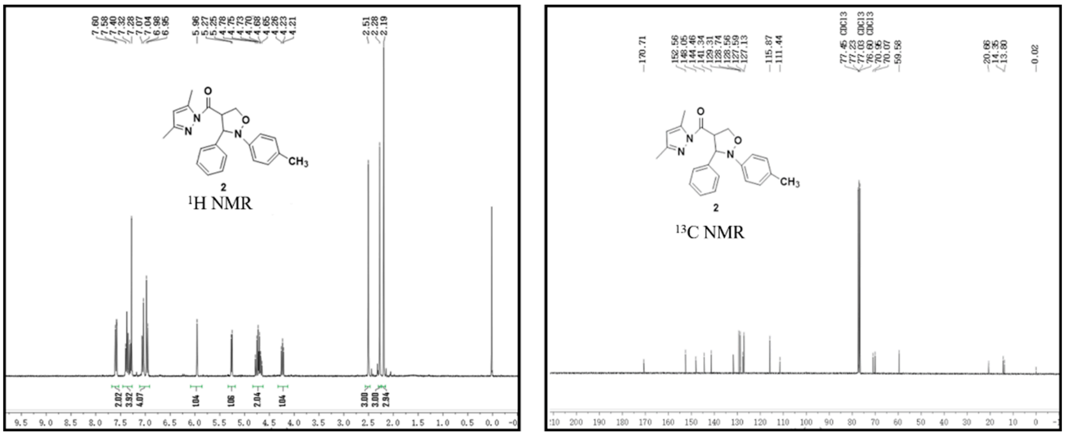

Figure S2. ^{1}H and ^{13}C NMR spectra of 4-(3,5-dimethylpyrazol-1-ylcarbonyl)-2-(4-methylphenyl)-3-phenylisoxazolidine (**2**) in $CDCl_3$

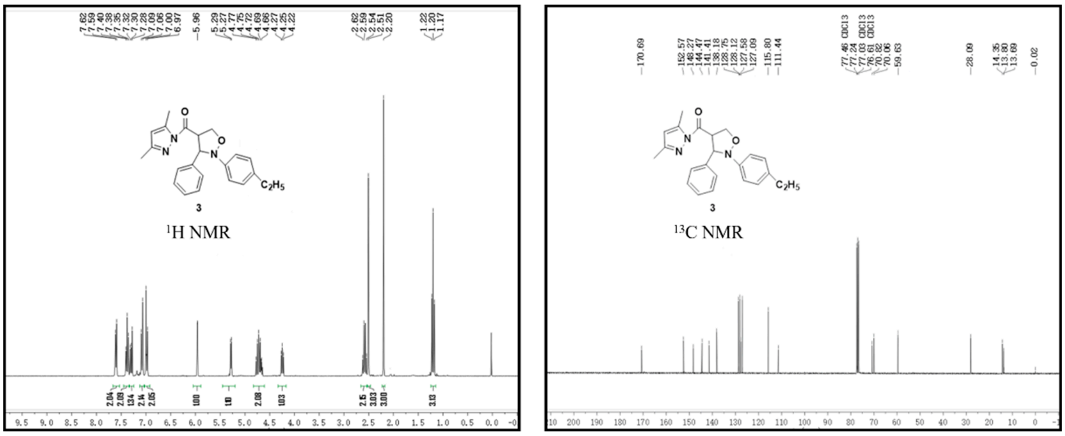

Figure S3. ^{1}H and ^{13}C NMR spectra of 4-(3,5-dimethylpyrazol-1-ylcarbonyl)-2-(4-ethylphenyl)-3-phenylisoxazolidine (**3**) in $CDCl_3$

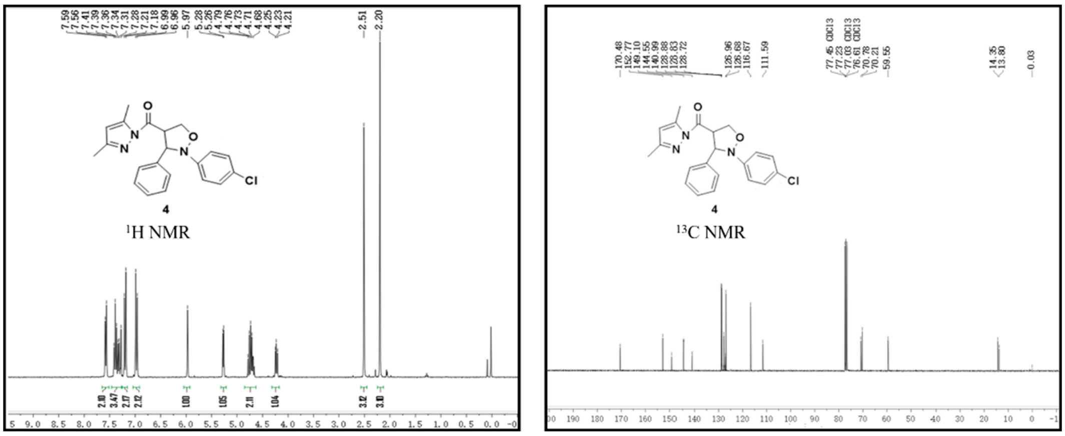

Figure S4. ^{1}H and ^{13}C NMR spectra of 4-(3,5-dimethylpyrazol-1-ylcarbonyl)-2-(4-chlorophenyl)-3-phenylisoxazolidine (**4**) in $CDCl_3$

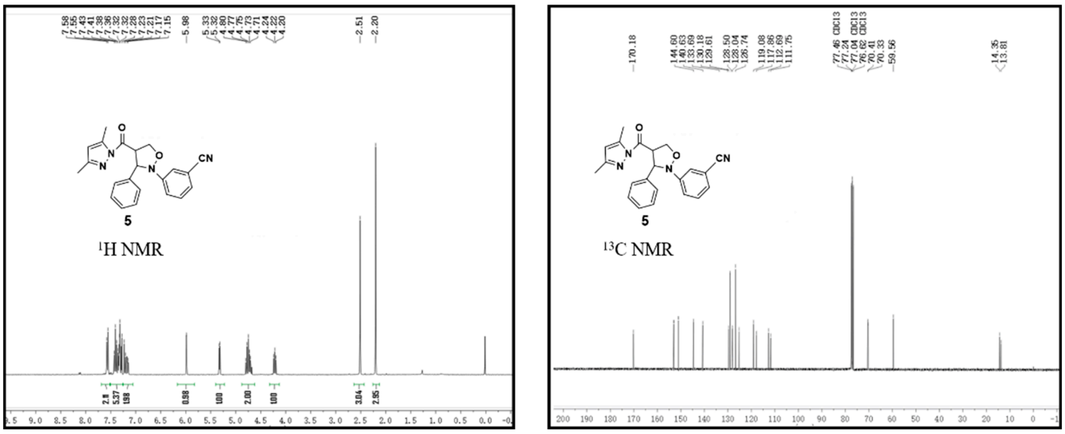

Figure S5. ^{1}H and ^{13}C NMR spectra of 4-(3,5-dimethylpyrazol-1-ylcarbonyl)-2-(3-cyanophenyl)-3-phenylisoxazolidine (**5**) in $CDCl_3$

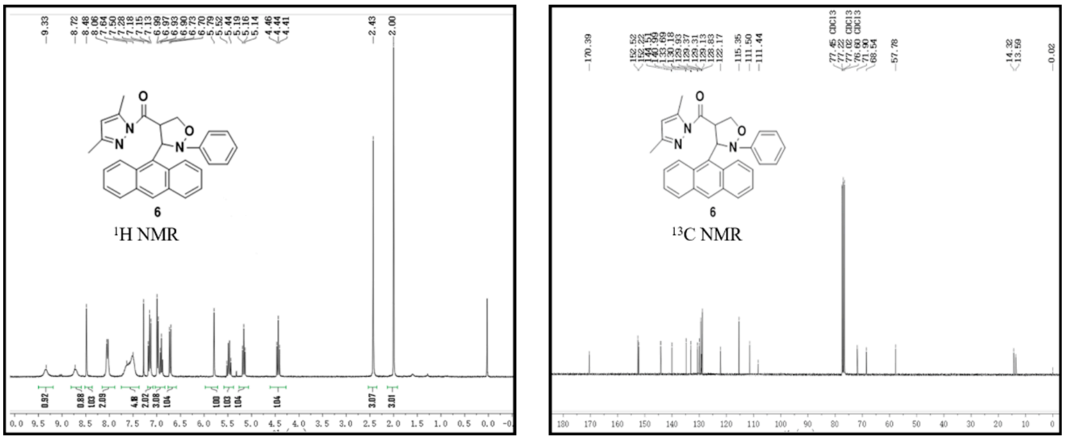

Figure S6. ^{1}H and ^{13}C NMR spectra of 3-(9-anthryl)-4-(3,5-dimethylpyrazol-1-ylcarbonyl)-2-phenylisoxazolidine (**6**) in $CDCl_3$

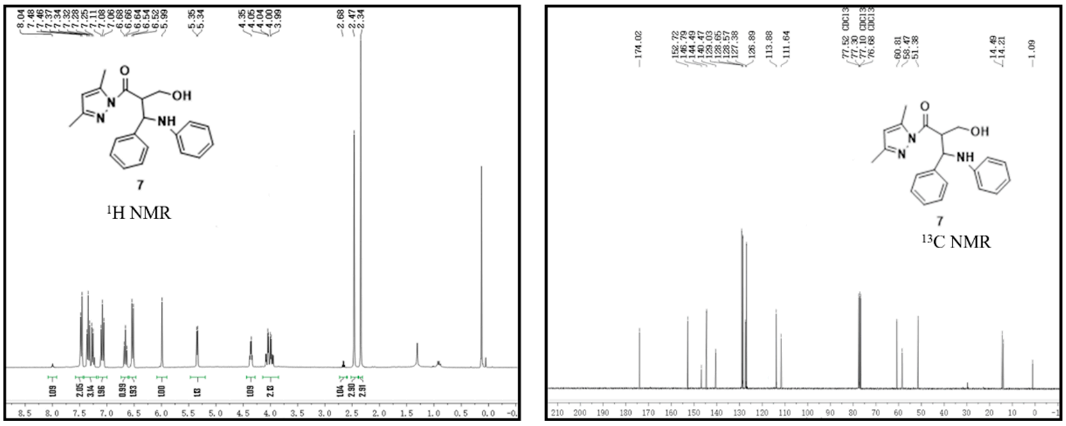

Figure S7. ^{1}H and ^{13}C NMR spectra of 2-(3,5-dimethylpyrazol-1-ylcarbonyl)-3-(phenylamino)-3-phenylpropan-1-ol (**7**) in $CDCl_3$

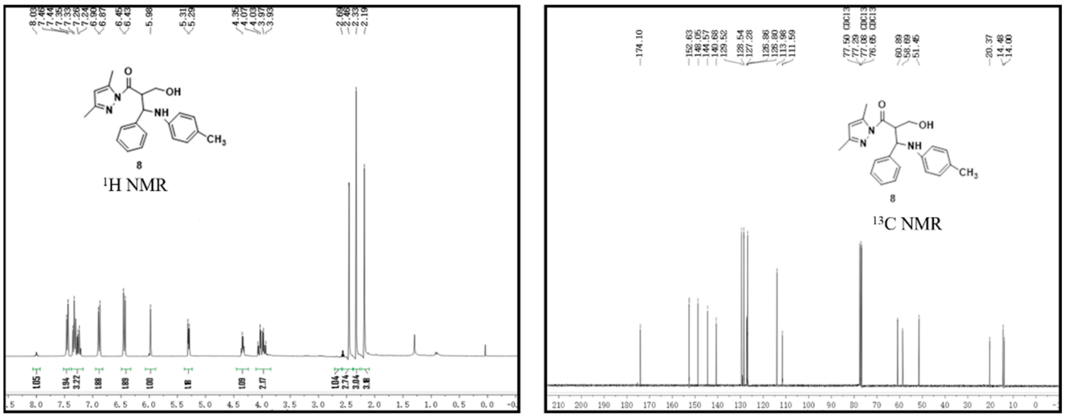

Figure S8. ^{1}H and ^{13}C NMR spectra of 2-(3,5-dimethylpyrazol-1-ylcarbonyl)-3-(4-methylphenylamino)-3-phenylpropan-1-ol (**8**) in $CDCl_3$

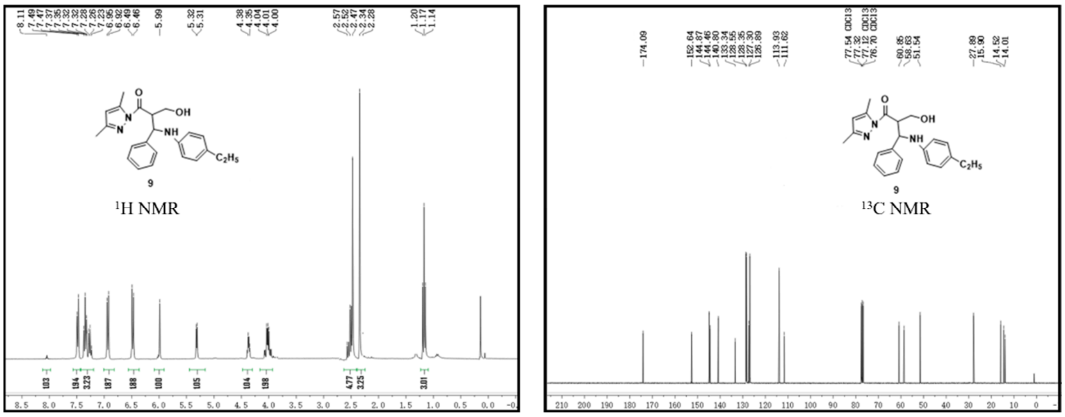

Figure S9. ^{1}H and ^{13}C NMR spectra of 2-(3,5-dimethylpyrazol-1-ylcarbonyl)-3-(4-ethylphenylamino)-3-phenylpropan-1-ol (**9**) in $CDCl_3$

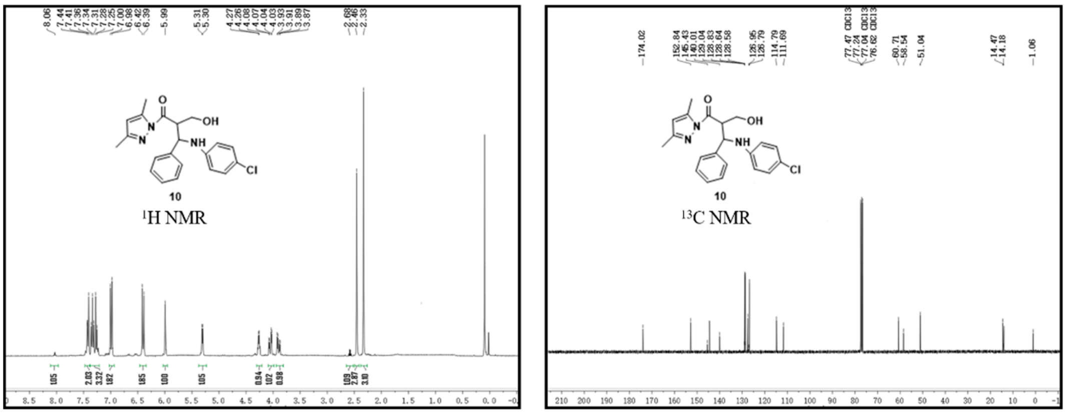

Figure S10. ^{1}H and ^{13}C NMR spectra of 2-(3,5-dimethylpyrazol-1-ylcarbonyl)-3-(4-chlorophenylamino)-3-phenylpropan-1-ol (**10**) in $CDCl_3$

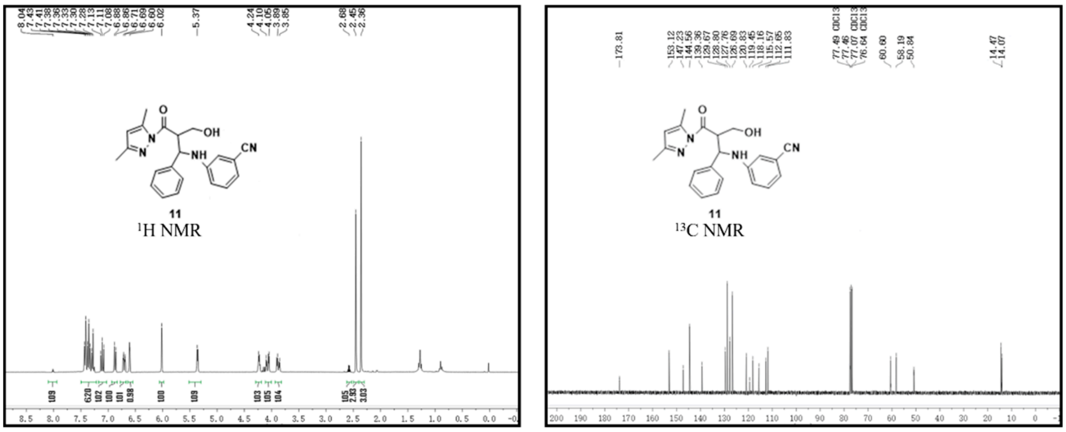

Figure S11. ^{1}H and ^{13}C NMR spectra of 2-(3,5-dimethylpyrazol-1-ylcarbonyl)-3-(3-cyanophenylamino)-3-phenylpropan-1-ol (**11**) in $CDCl_3$

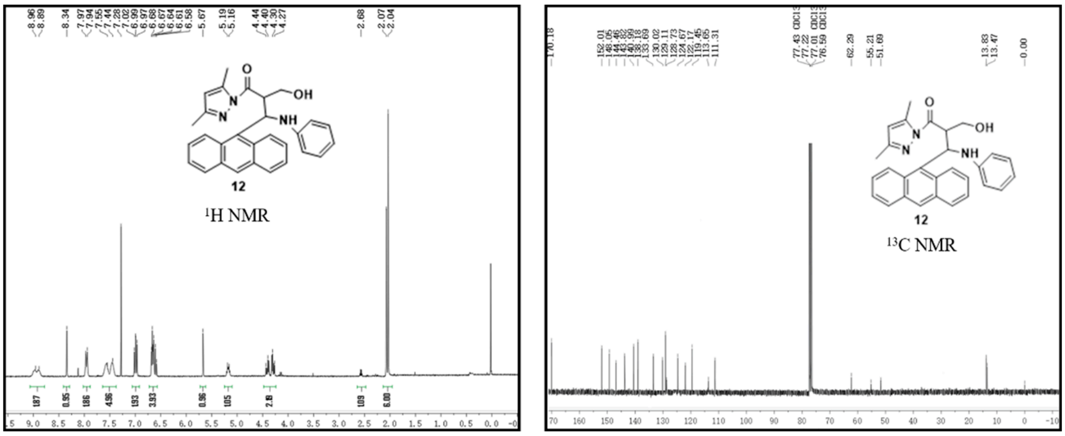

Figure S12. ^{1}H and ^{13}C NMR spectra of 2-(3,5-dimethylpyrazol-1-ylcarbonyl)-3-(phenylamino)-3-(9-anthryl)propan-1-ol (**12**) in $CDCl_3$

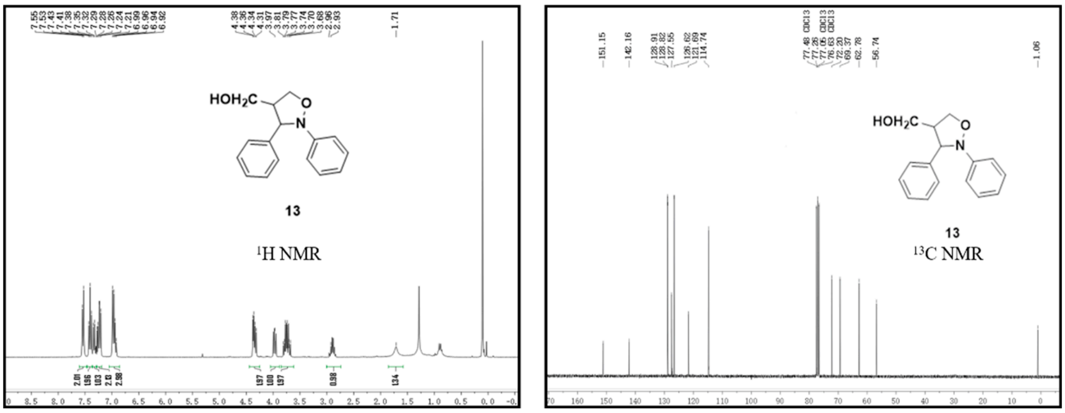

Figure S13. ^{1}H and ^{13}C NMR spectra of 4-hydroxymethyl-2,3-diphenylisoxazolidine (**13**) in $CDCl_3$

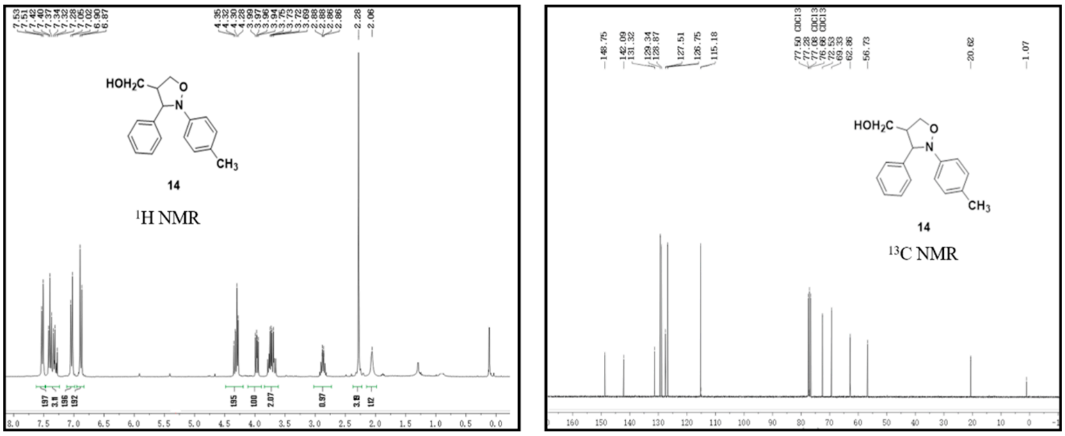

Figure S14. ^{1}H and ^{13}C NMR spectra of 2-(4-methylphenyl)-4-hydroxymethyl-3-phenylisoxazolidine (**14**) in $CDCl_3$

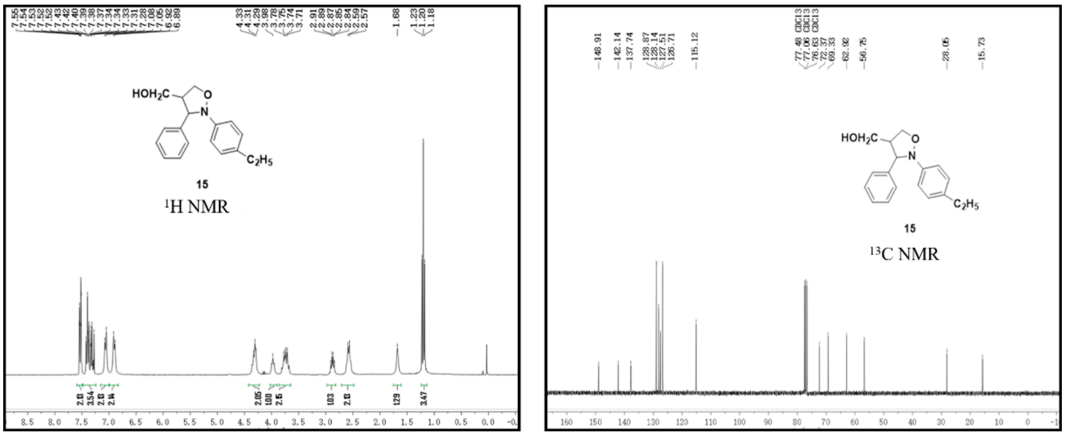

Figure S15. ^{1}H and ^{13}C NMR spectra of 2-(4-ethylphenyl)-4-hydroxymethyl-3-phenylisoxazolidine (**15**) in $CDCl_3$

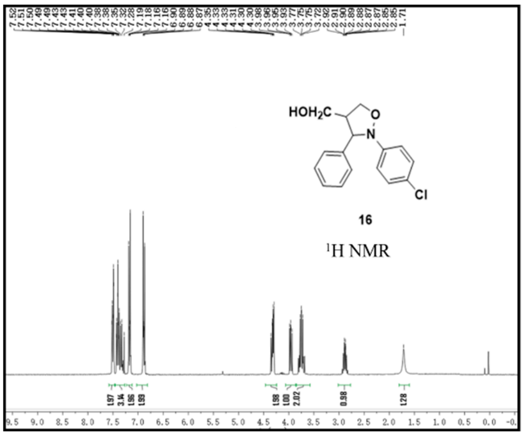

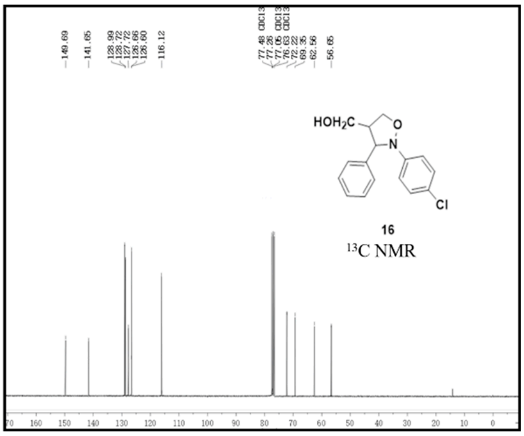

Figure S16. ^{1}H and ^{13}C NMR spectra of 2-(4-chlorophenyl)-4-hydroxymethyl-3-phenylisoxazolidine (**16**) in $CDCl_3$

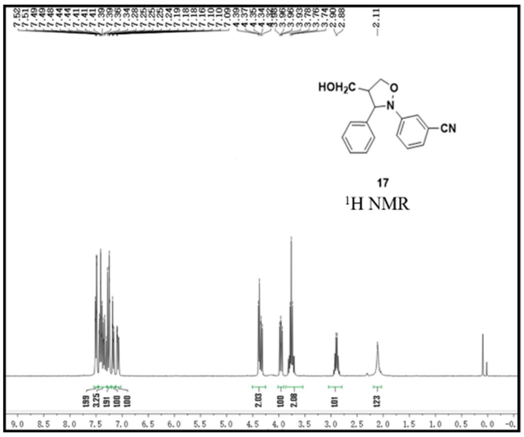

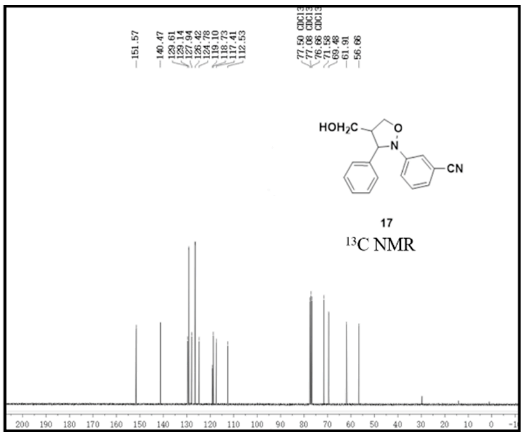

Figure S17. ^{1}H and ^{13}C NMR spectra of 2-(3-cyanophenyl)-4-hydroxymethyl-3-phenylisoxazolidine (**17**) in $CDCl_3$

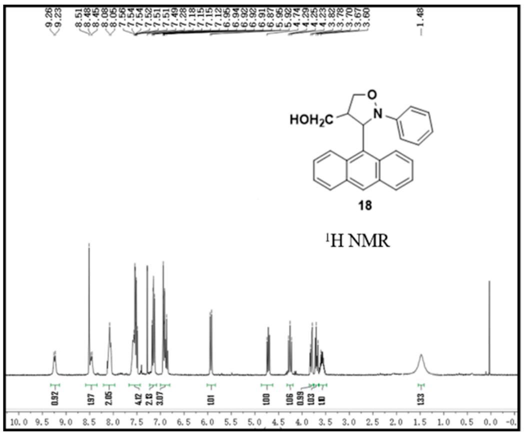

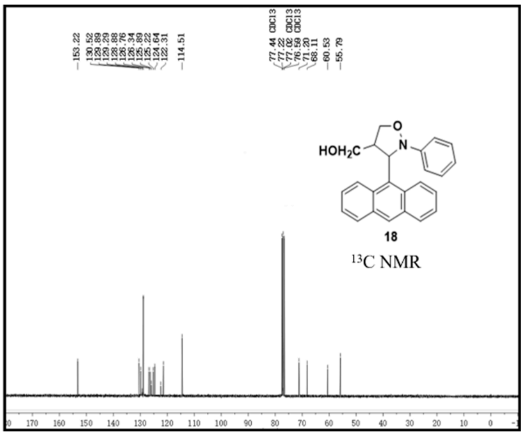

Figure S18. ^{1}H and ^{13}C NMR spectra of 3-(9-anthryl)-4-hydroxymethyl-2-phenylisoxazolidine (**18**) in $CDCl_3$

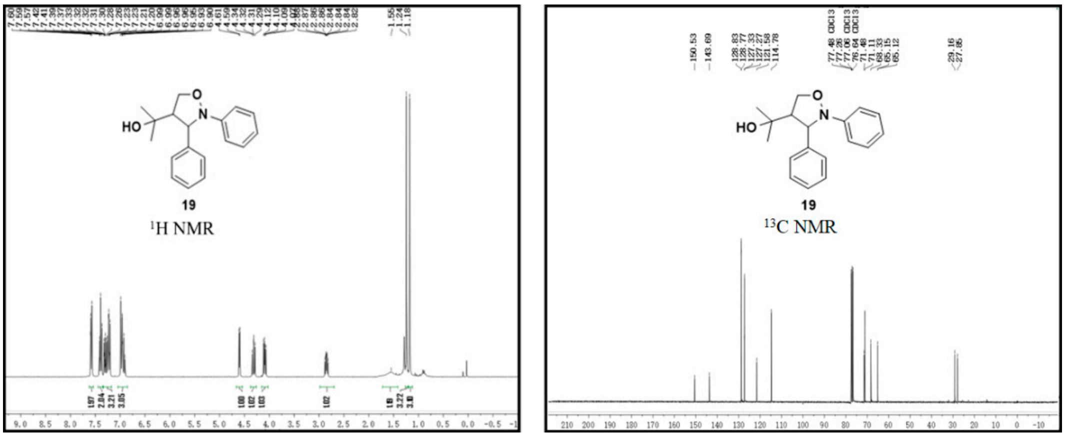

Figure S19. ^{1}H and ^{13}C NMR spectra of 2-(2-(4-phenyl)-3-phenylisoxazolidin-4-yl)propan-2-ol (**19**) in $CDCl_3$

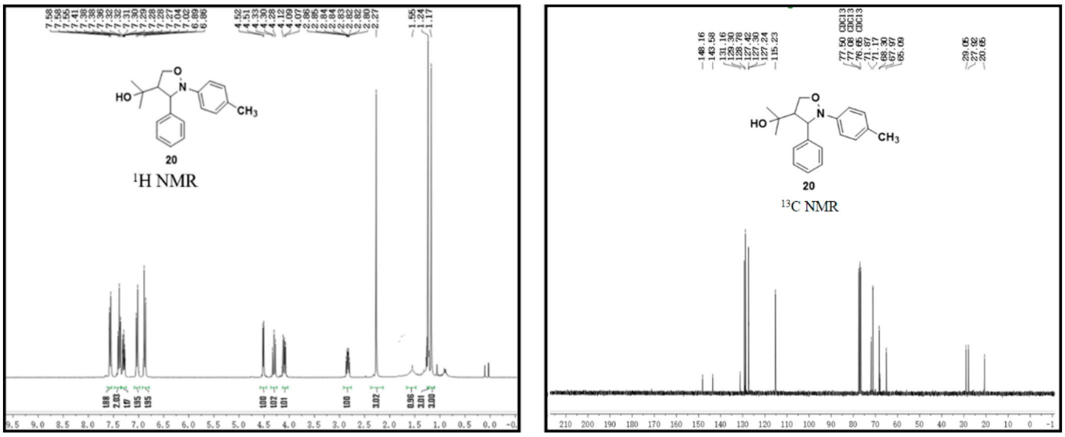

Figure S20. ^{1}H and ^{13}C NMR spectra of 2-(2-(4-methylphenyl)-3-phenylisoxazolidin-4-yl)propan-2-ol (**20**) in $CDCl_3$

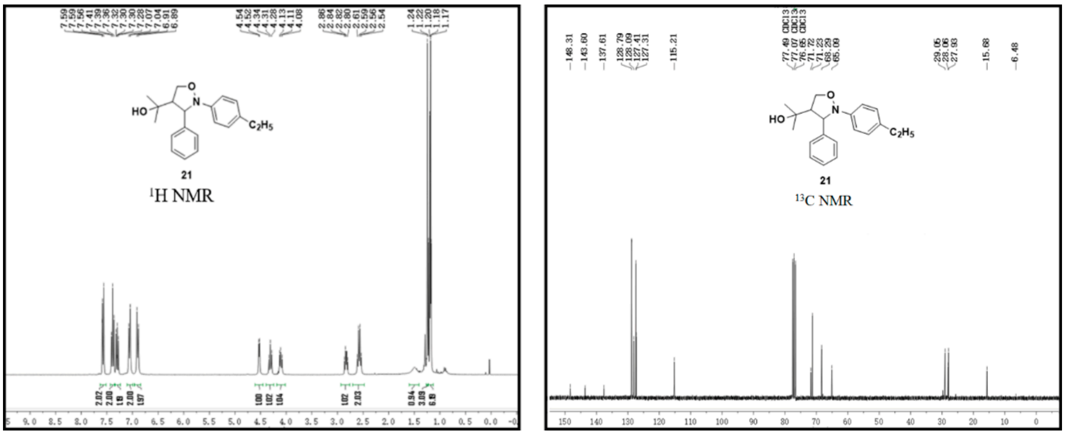

Figure S21. ^{1}H and ^{13}C NMR spectra of 2-(2-(4-ethylphenyl)-3-phenylisoxazolidin-4-yl)propan-2-ol (**21**) in $CDCl_3$

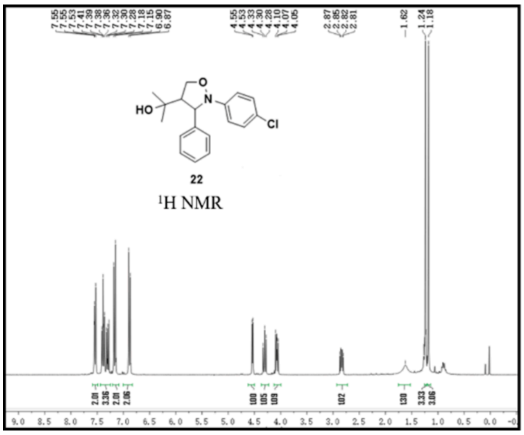

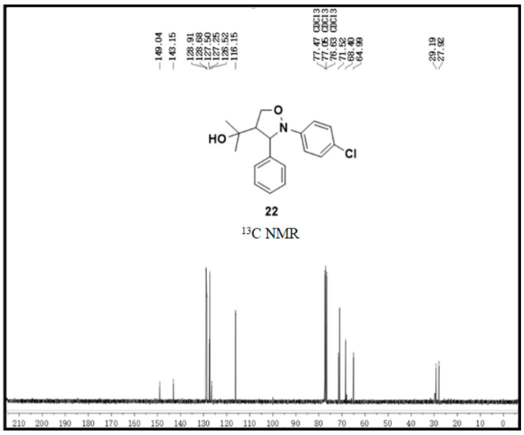

Figure S22. ^{1}H and ^{13}C NMR spectra of 2-(2-(4-chlorophenyl)-3-phenylisoxazolidin-4-yl)propan-2-ol (**22**) in $CDCl_3$

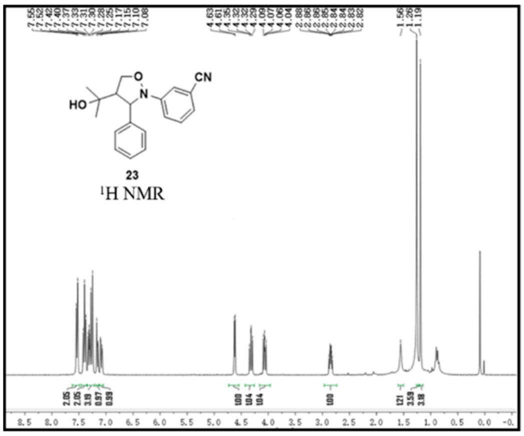

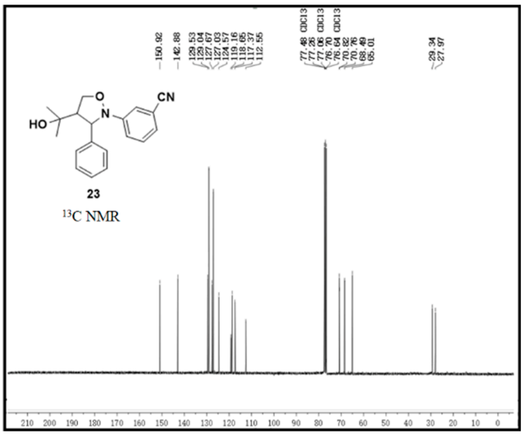

Figure S23. ^{1}H and ^{13}C NMR spectra of 2-(2-(3-cyanophenyl)-3-phenylisoxazolidin-4-yl)propan-2-ol (**23**) in $CDCl_3$

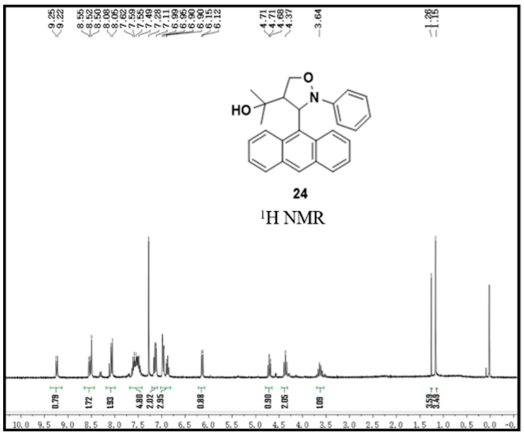

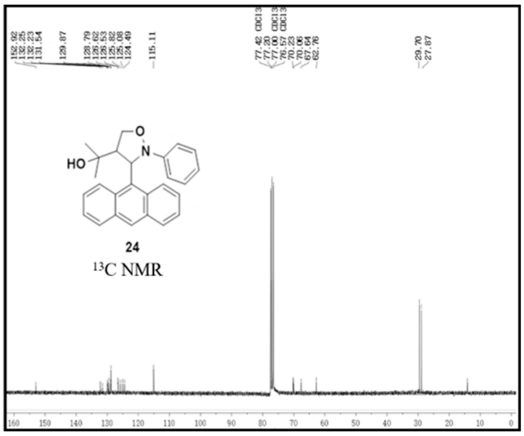

Figure S24. ^{1}H and ^{13}C NMR spectra of 2-(2-(3-phenyl)-3-(9-anthryl)isoxazolidin-4-yl)propan-2-ol (**24**) in $CDCl_3$

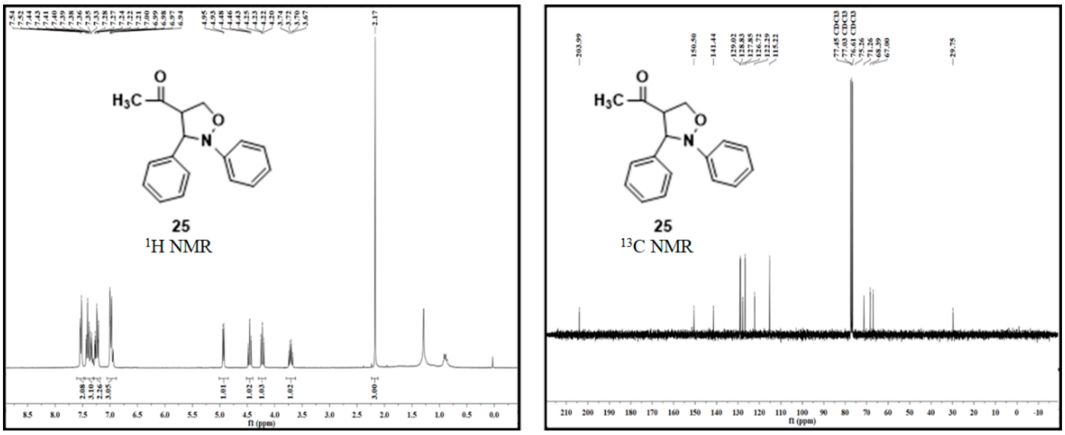

Figure S25. ^{1}H and ^{13}C NMR spectra of 4-acetyl-2,3-diphenylisoxazolidine (**25**) in $CDCl_3$

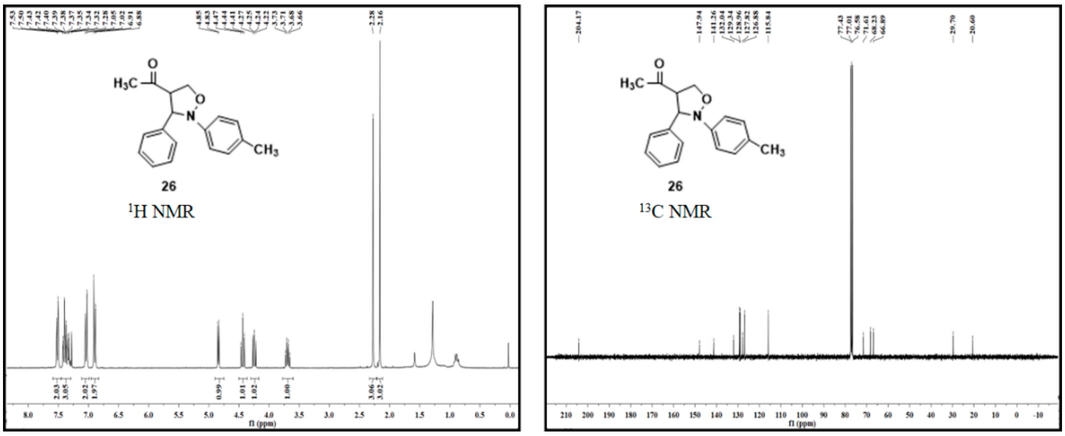

Figure S26. ^{1}H and ^{13}C NMR spectra of 4-acetyl-2-(4-methylphenyl)-3-phenylisoxazolidine (**26**) in $CDCl_3$

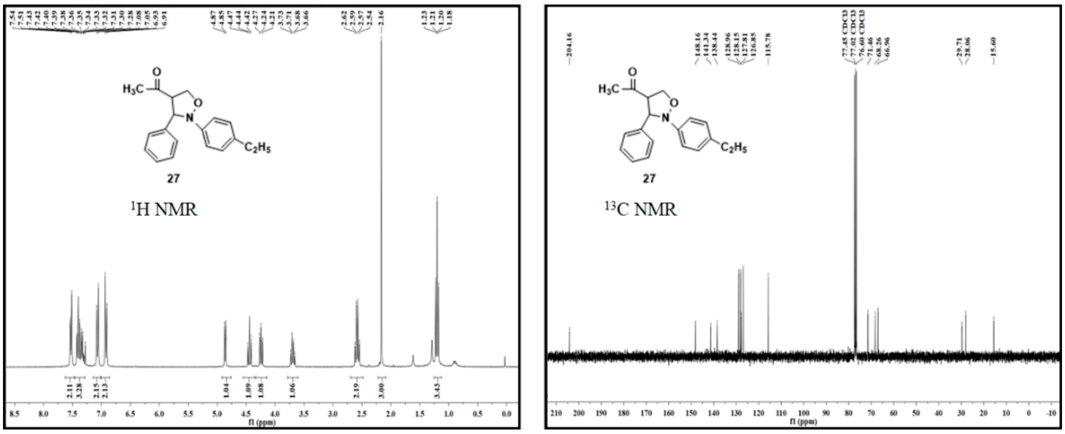

Figure S27. ^{1}H and ^{13}C NMR spectra of 4-acetyl-2-(4-ethylphenyl)-3-phenylisoxazolidine (**27**) in $CDCl_3$

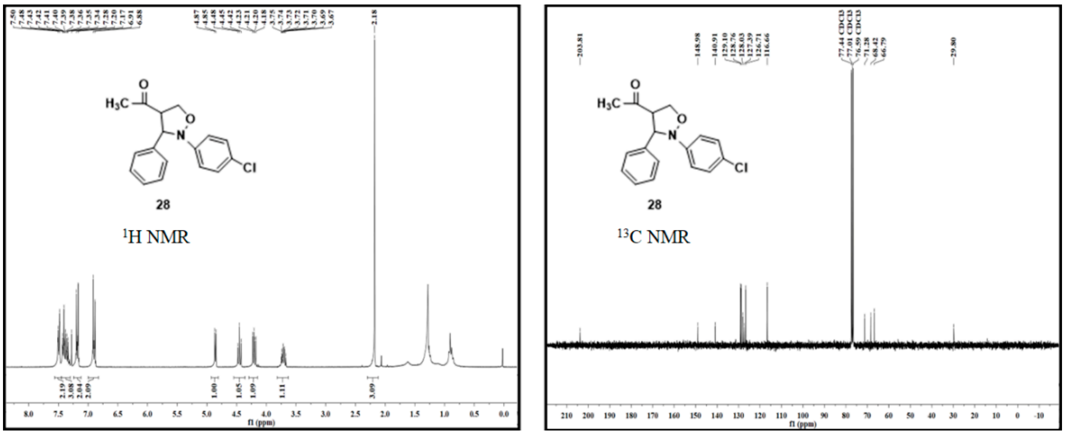

Figure S28. ^{1}H and ^{13}C NMR spectra of 4-acetyl-2-(4-chlorophenyl)-3-phenylisoxazolidine (**28**) in $CDCl_3$

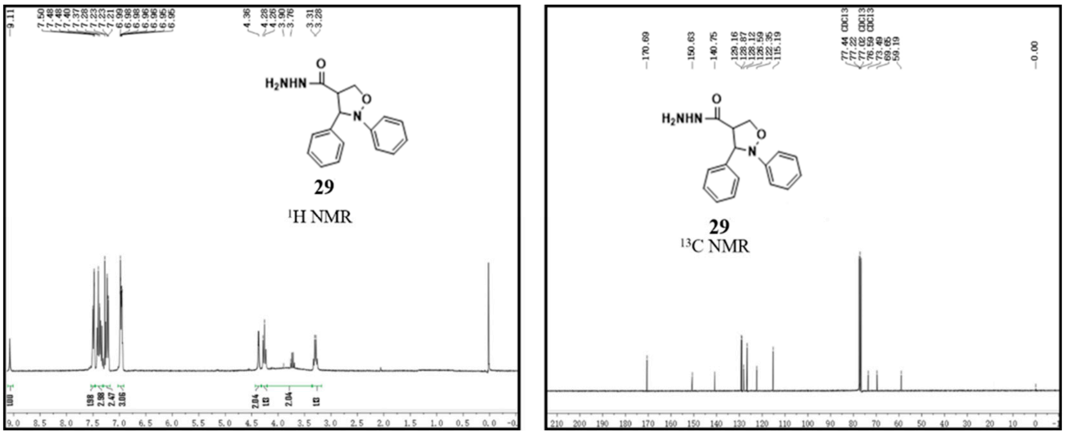

Figure S29. ^{1}H and ^{13}C NMR spectra of 2,3-diphenylisoxazolidin-4-ylcarbohydrazide (**29**) in $CDCl_3$

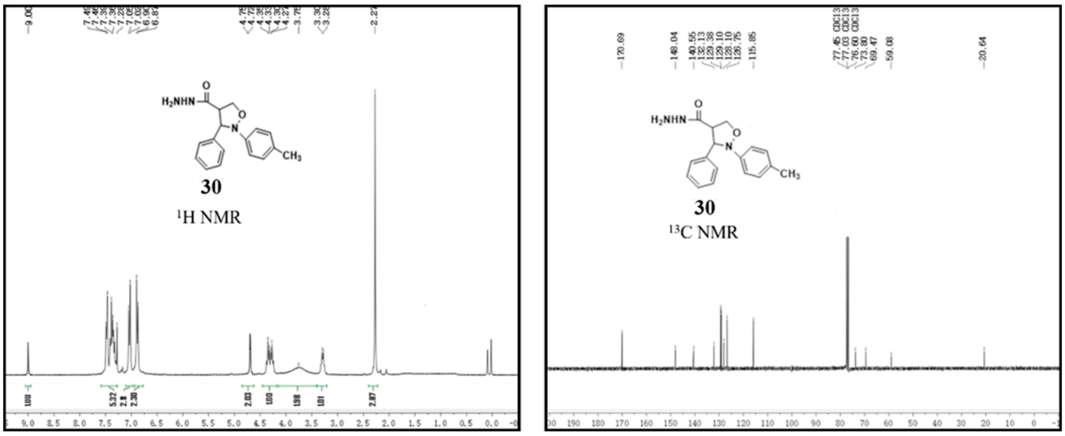

Figure S30. ^{1}H and ^{13}C NMR spectra of 2-(4-methylphenyl)-3-phenylisoxazolidin-4-ylcarbohydrazide (**30**) in $CDCl_3$

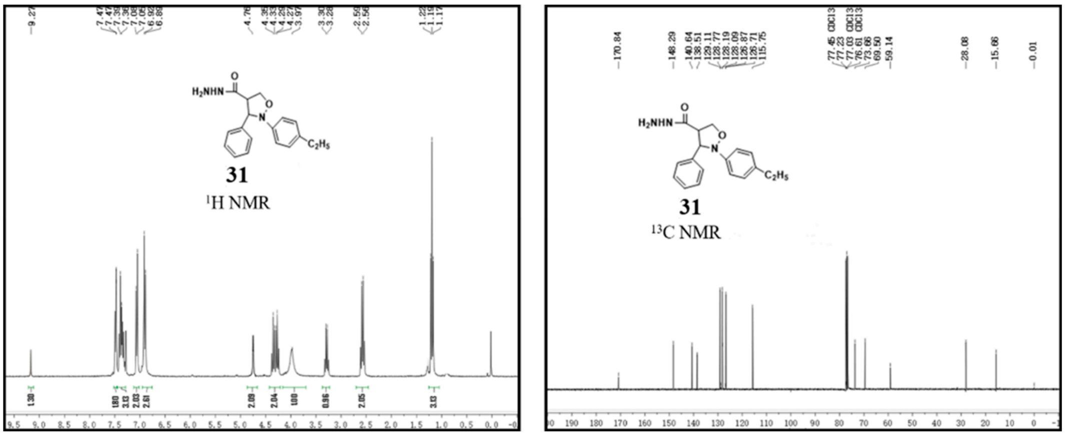

Figure S31. ^{1}H and ^{13}C NMR spectra of 2-(4-ethylphenyl)-3-phenylisoxazolidin-4-ylcarbohydrazide (**31**) in $CDCl_3$

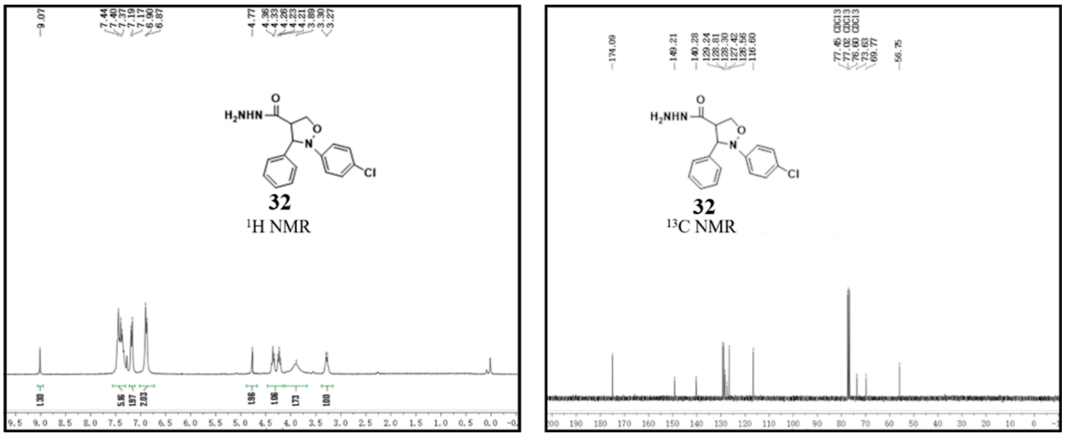

Figure S32. ^{1}H and ^{13}C NMR spectra of 2-(4-chlorophenyl)-3-phenylisoxazolidin-4-ylcarbohydrazide (**32**) in $CDCl_3$

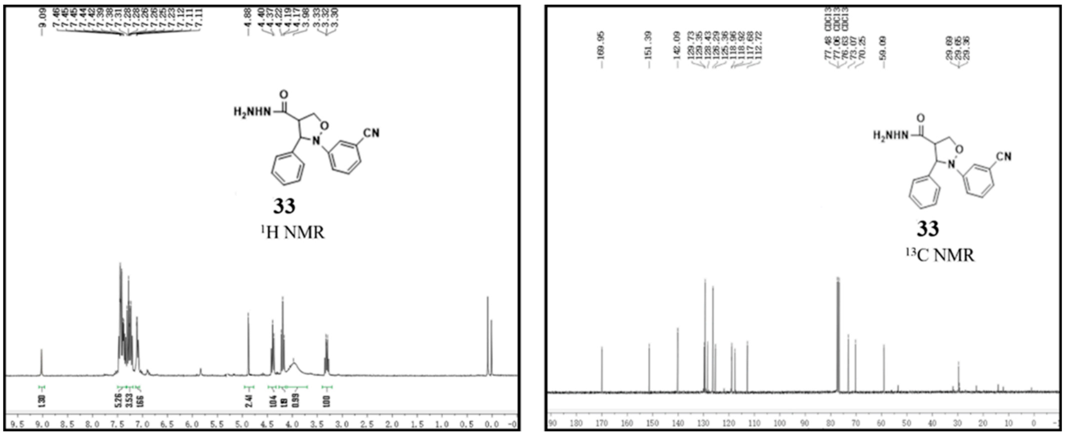

Figure S33. ^{1}H and ^{13}C NMR spectra of 2-(3-cyanophenyl)-3-phenylisoxazolidin-4-ylcarbohydrazide (**33**) in $CDCl_3$

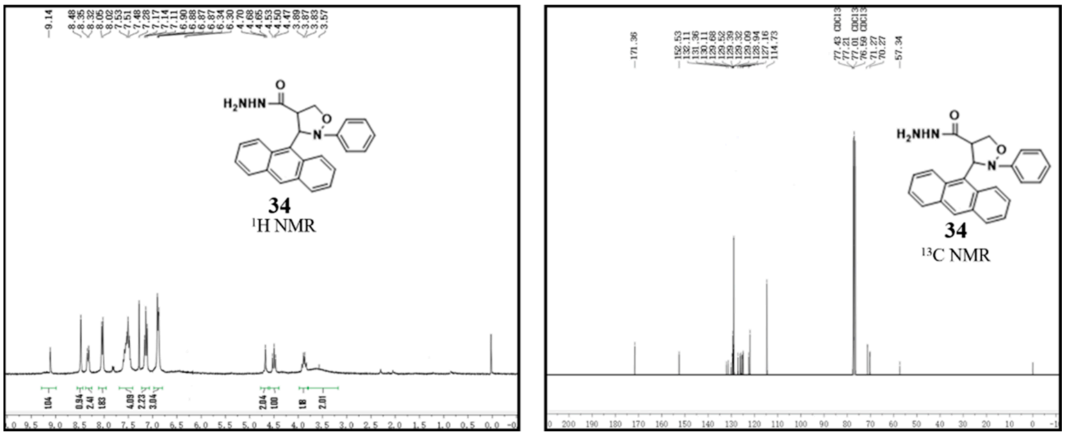

Figure S34. ^{1}H and ^{13}C NMR spectra of 2-phenyl-3-(9-anthryl)isoxazolidin-4-ylcarbohydrazide (**34**) in $CDCl_3$

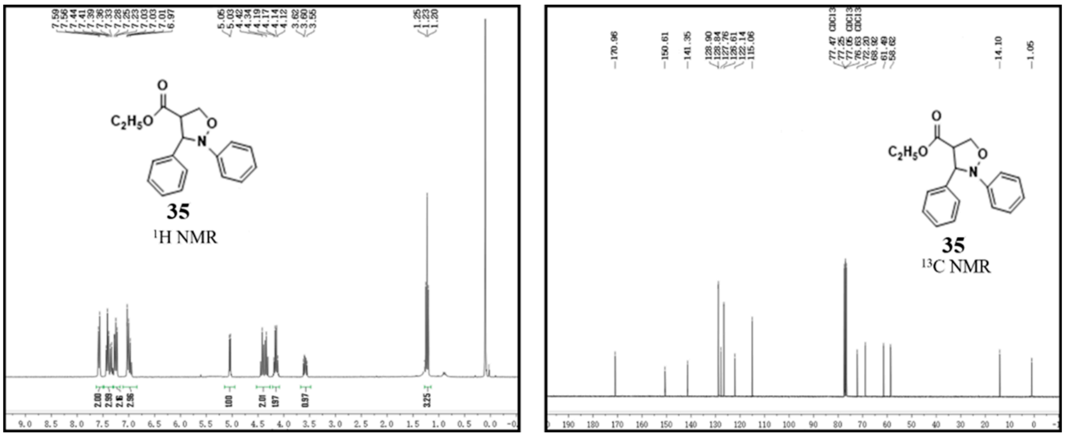

Figure S35. ^{1}H and ^{13}C NMR spectra of ethyl 2,3-diphenylisoxazolidin-4-ylcarboxylate (**35**) in $CDCl_3$

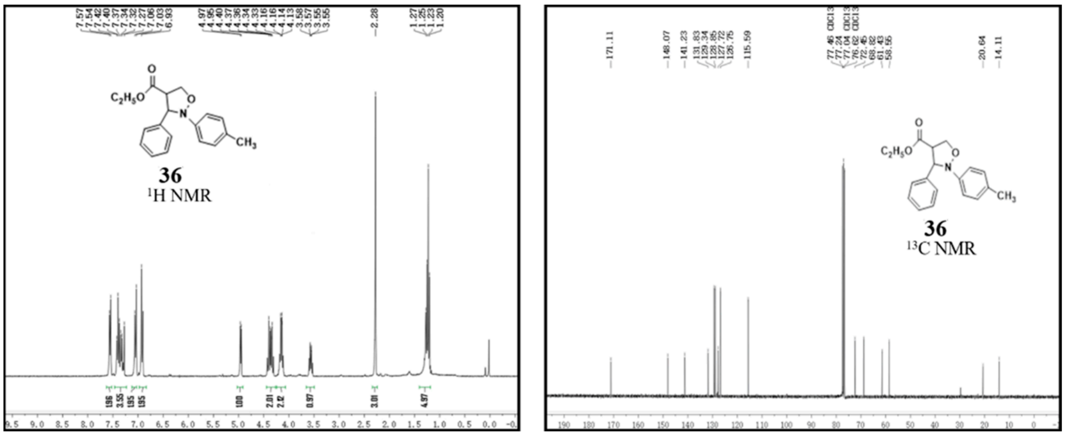

Figure S36. ^{1}H and ^{13}C NMR spectra of ethyl 2-(4-methylphenyl)-3-phenylisoxazolidin-4-ylcarboxylate (**36**) in $CDCl_3$

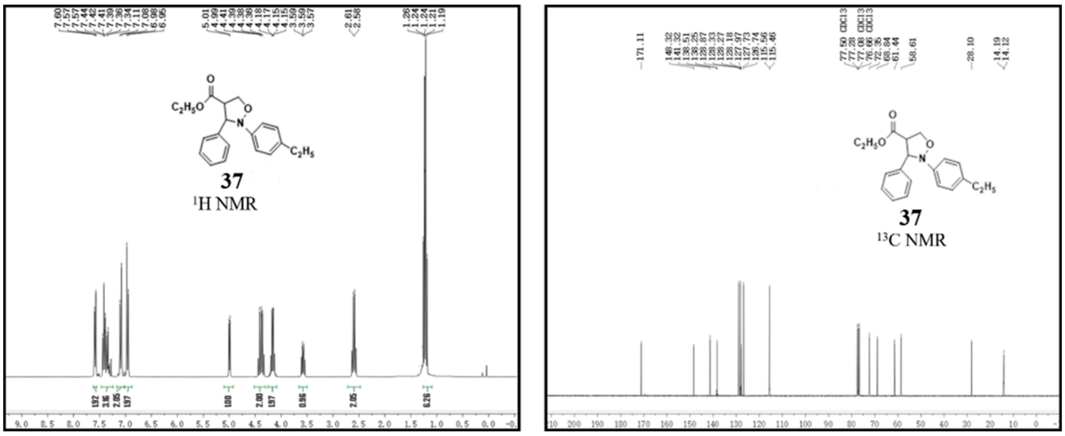

Figure S37. ^{1}H and ^{13}C NMR spectra of ethyl 2-(4-ethylphenyl)-3-phenylisoxazolidin-4-ylcarboxylate (**37**) in $CDCl_3$

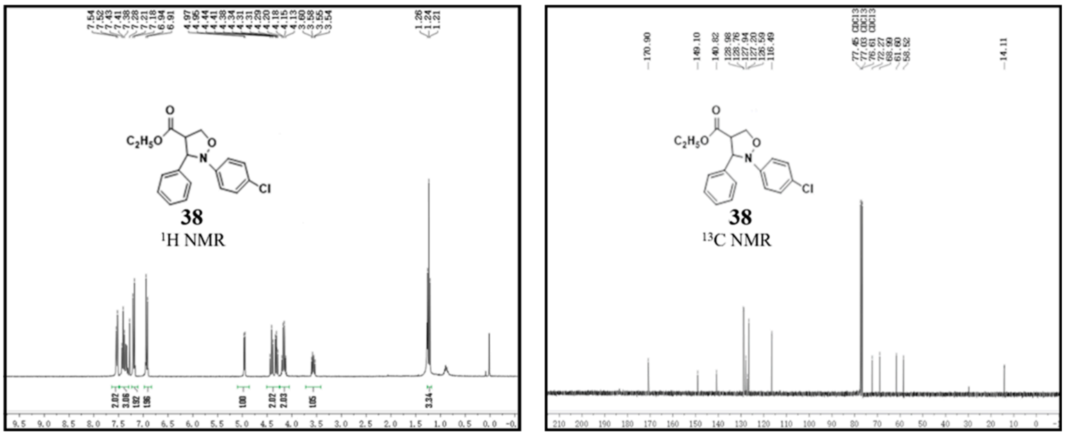

Figure S38. ^{1}H and ^{13}C NMR spectra of ethyl 2-(4-chlorophenyl)-3-phenylisoxazolidin-4-ylcarboxylate (**38**) in $CDCl_3$

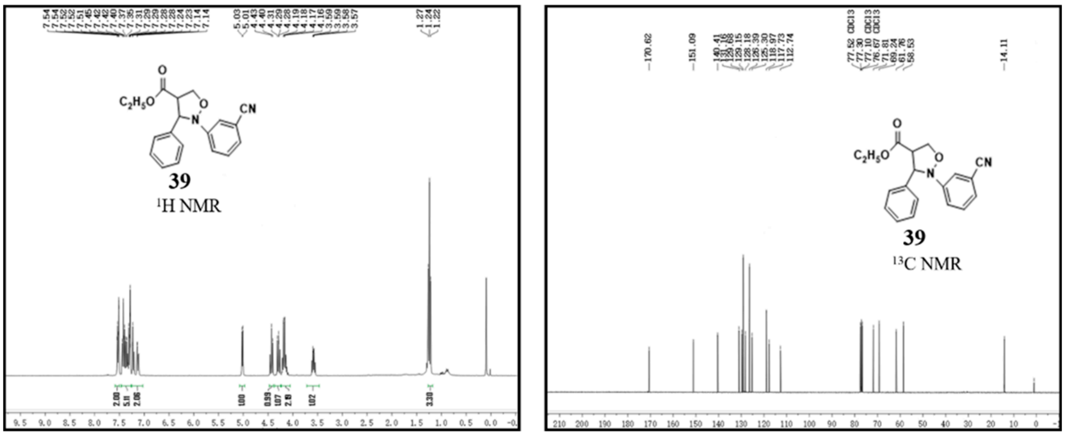

Figure S39. ^{1}H and ^{13}C NMR spectra of ethyl 2-(3-cyanophenyl)-3-phenylisoxazolidin-4-ylcarboxylate (**39**) in $CDCl_3$

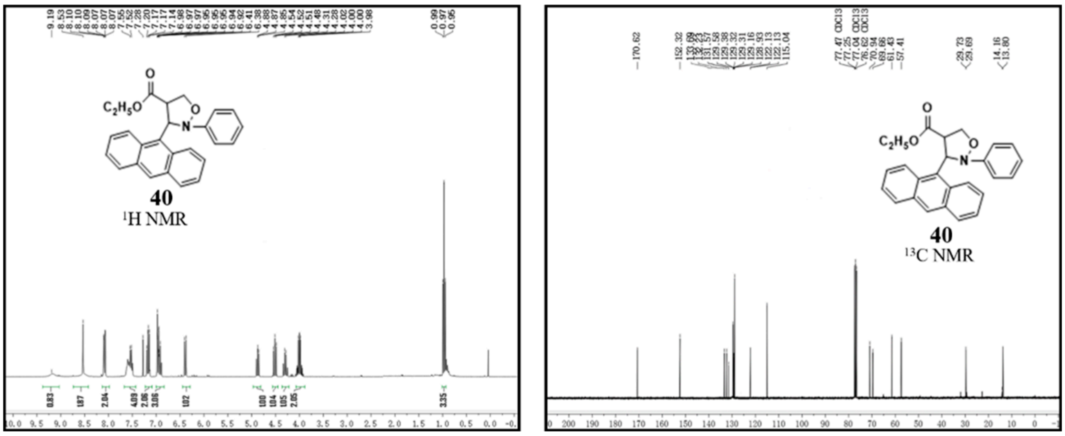

Figure S40. ^{1}H and ^{13}C NMR spectra of ethyl 3-(9-anthryl)-2-phenylisoxazolidin-4-ylcarboxylate (**40**) in $CDCl_3$

3. Crystallographic data of compound **6**.

Table S1. Crystal data and structure refinement for compound **6**.

Compound	**6**
CCDC number	2234690
molecular formula	$C_{29}H_{25}N_3O_2$
formula weight	447.52
Temperature/K	169.99(10)
crystal system	triclinic
space group	P-1
a (Å)	9.4969(7)
b (Å)	10.4040(9)
c (Å)	13.0319(10)
α (°)	92.175(7)
β (°)	101.420(7)
γ (°)	117.088(8)
V (Å^3)	1111.51(17)
Z	2
D_c (g·cm^{-3})	1.337
μ (mm^{-1})	0.675
F(000)	472.0
Crystal size/mm^3	0.13 × 0.12 × 0.1
Index ranges	$-11 \leq h \leq 11$<br>$-12 \leq k \leq 12$<br>$-15 \leq l \leq 16$
R_1[a] [I > 2σ(I)]	0.0658
wR_2[b] (all data)	0.1896
GOF on F^2	1.081
Largest diff. peak/hole / e Å^{-3}	0.56/-0.43

Table S2. Selected bond distances (Å) and angles (°) for compound **6**.

Compound **6**			
O(1)-N(1)	1.483(3)	N(2)-C(22)	1.312(4)
O(1)-C(18)	1.429(4)	N(3)-C(19)	1.399(4)
O(2)-C(19)	1.210(4)	N(3)-C(20)	1.392(4)
N(1)-C(17)	1.481(3)	C(1)-C(2)	1.497(4)
N(1)-C(25)	1.446(4)	C(1)-C(6)	1.417(4)
N(2)-N(3)	1.384(4)	C(1)-C(14)	1.417(4)
C(18)-O(1)-N(1)	101.87(19)	N(2)-N(3)-C(19)	119.2(2)
C(17)-N(1)-O(1)	101.08(19)	N(2)-N(3)-C(20)	111.1(2)
C(25)-N(1)-O(1)	105.3(2)	C(20)-N(3)-C(19)	129.7(3)
C(25)-N(1)-C(17)	116.3(2)	C(6)-C(1)-C(2)	117.0(3)
C(22)-N(2)-N(3)	105.1(3)	C(14)-C(1)-C(2)	122.8(3)

4. Reference

[1] Li, M.; Cao, X.; You, J.; Yu, Y.; Wu, W.; Liu, B. Asymmetric 1,3-Dipolar Cycloaddition Reaction of *C,N*-Diarylnitrone with *N*-*α*,*β*-Unsaturated Acyl Compounds Catalyzed by Chiral Bisoxazoline Metal Complex. *Chin, J. Org. Chem.* **2019**, *39*, 1642–1649.
